# From primordial clocks to circadian oscillators

**DOI:** 10.1038/s41586-023-05836-9

**Published:** 2023-03-22

**Authors:** Warintra Pitsawong, Ricardo A. P. Pádua, Timothy Grant, Marc Hoemberger, Renee Otten, Niels Bradshaw, Nikolaus Grigorieff, Dorothee Kern

**Affiliations:** 1 Howard Hughes Medical Institute and Department of Biochemistry, Brandeis University, Waltham, MA USA; 2 Janelia Research Campus, Howard Hughes Medical Institute, Ashburn, VA USA; 3 Department of Biochemistry, Brandeis University, Waltham, MA USA; 4 Present Address: Biomolecular Discovery, Relay Therapeutics, Cambridge, MA USA; 5 Present Address: John and Jeanne Rowe Center for Research in Virology, Morgridge Institute for Research, Madison, Madison, WI USA; 6 Present Address: Department of Biochemistry, University of Wisconsin-Madison, Madison, WI USA; 7 Present Address: Treeline Biosciences, Watertown, MA USA; 8 Present Address: Howard Hughes Medical Institute, RNA Therapeutics Institute, University of Massachusetts Chan Medical School, Worcester, MA USA

**Keywords:** Enzyme mechanisms, Structural biology

## Abstract

Circadian rhythms play an essential part in many biological processes, and only three prokaryotic proteins are required to constitute a true post-translational circadian oscillator^1^. The evolutionary history of the three Kai proteins indicates that KaiC is the oldest member and a central component of the clock^2^. Subsequent additions of KaiB and KaiA regulate the phosphorylation state of KaiC for time synchronization. The canonical KaiABC system in cyanobacteria is well understood^3–6^, but little is known about more ancient systems that only possess KaiBC. However, there are reports that they might exhibit a basic, hourglass-like timekeeping mechanism^7–9^. Here we investigate the primordial circadian clock in *Rhodobacter sphaeroides*, which contains only KaiBC, to elucidate its inner workings despite missing KaiA. Using a combination of X-ray crystallography and cryogenic electron microscopy, we find a new dodecameric fold for KaiC, in which two hexamers are held together by a coiled-coil bundle of 12 helices. This interaction is formed by the carboxy-terminal extension of KaiC and serves as an ancient regulatory moiety that is later superseded by KaiA. A coiled-coil register shift between daytime and night-time conformations is connected to phosphorylation sites through a long-range allosteric network that spans over 140 Å. Our kinetic data identify the difference in the ATP-to-ADP ratio between day and night as the environmental cue that drives the clock. They also unravel mechanistic details that shed light on the evolution of self-sustained oscillators.

## Abstract (web summary)

X-ray, cryo-EM and kinetic studies of the circadian oscillator KaiBC from the photosynthetic bacterium *Rhodobacter sphaeroides* shed light on the evolution of self-regulating oscillators.

## Main

Circadian clocks are self-sustained biological oscillators that are ubiquitously found in prokaryotic and eukaryotic organisms. In eukaryotes, these systems are complex and highly sophisticated, whereas in prokaryotes, the core mechanism is regulated by a post-translational oscillator that can be reconstituted in vitro with ATP and three proteins (encoded by *kaiA*, *kaiB* and *kaiC*)^1^. Seminal work on the KaiABC system has resulted in a comprehensive understanding of its circadian clock. KaiC is the central component that autophosphorylates by binding to KaiA and autodephosphorylates following association with KaiB^3–6^. The interplay among these three proteins has been shown in vitro to constitute a true circadian oscillator characterized by persistence, resetting and temperature compensation. Consequently, the KaiABC system is considered an elegant and the simplest implementation of a circadian rhythm. The evolutionary history of *kai* genes established *kaiC* as the oldest member dating back around 3.5 billion years ago. Subsequent additions of *kaiB* and most recently *kaiA* formed the extant *kaiBC* and *kaiABC* clusters, respectively^2,10^. Notably, some studies of more primitive organisms that lack *kaiA* hinted that the *kaiBC*-based systems might already provide a basic, hourglass-like timekeeping mechanism^7–9^. Contrary to the self-sustained oscillators found in cyanobacteria, such a timer requires an environmental cue to drive the clock and for the daily flip of the hourglass. The central role of circadian rhythms in many biological processes, controlled by the day and night cycle on Earth, makes their evolution a fascinating topic.

Here we investigate such a primitive circadian clock through biochemical and structural studies of the KaiBC system of the purple, nonsulfur photosynthetic proteobacterium *R.* *sphaeroides* KD131 (hereafter, its components are referred to as KaiB_RS_ and KaiC_RS_). The organism shows sustained rhythms of gene expression in vivo, but whether *kaiBC* is responsible for this observation remains inconclusive in the absence of a *kaiC* knockout^11^. A previous study of the closely related bacterium *Rhodopseudomonas palustris* that used a knockout strain demonstrated causality between the proto-circadian rhythm of nitrogen fixation and expression of the *kaiC* gene^9^. Here through in vitro experiments, we discover that KaiBC_RS_ is a primordial circadian clock with a mechanism that is different from the widely studied circadian oscillator in *Synechococcus elongatus* PCC 7942 (hereafter, its components are referred to as KaiA_SE_, KaiB_SE_ and KaiC_SE_)^3–6^. We identify an environmental cue that regulates the phosphorylation state and consequently produces a 24 h clock in vivo as the switch in the ATP-to-ADP ratio between day and night. Our results from kinetic studies combined with X-ray and cryogenic electron microscopy (cryo-EM) structures of the relevant states unravel a long-range allosteric pathway that is crucial for the function of the hourglass and sheds light on the evolution of self-sustained oscillators. Notably, we find a new protein fold for KaiC_RS_ and uncover a register shift in the coiled-coil domain that spans around 115 Å as the key regulator in this system, which shows structural similarities to dynein signalling^12^.

## The C-terminal tail is a primitive regulatory moiety

**Fig. 1:**
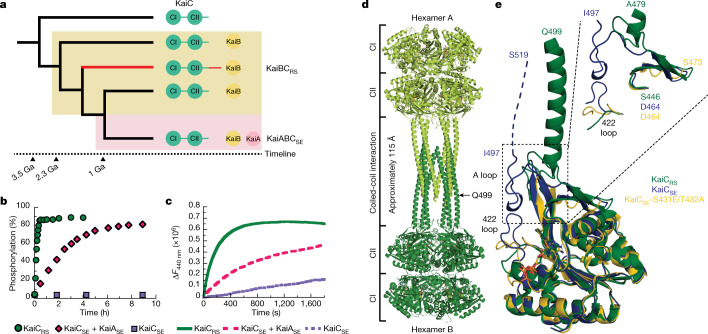
The extended C-terminal tail of KaiC_RS_ forms a coiled-coil interaction with an exposed A loop for KaiA-independent phosphorylation of KaiC. **a**, Schematic of the phylogenetic tree of *kaiC* showing the appearance of *kaiB* and *kaiA* during evolution. The *kaiC* clade with an approximately 50-amino-acid C-terminal extension is labelled in red, and a timeline was predicted as previously reported^2^. Ga, billion years ago. **b**, Phosphorylation rate over time of KaiC_RS_ (6.5 ± 1.0 h^−1^) and KaiC_SE_ in the presence (0.40 ± 0.02 h^−1^) or absence of KaiA_SE_ at 30 °C. The s.d. in reported parameters were obtained from the fitting. **c**, Nucleotide exchange between ATP and mant-ATP in KaiC_RS_ alone (18.0 ± 1.5 h^–1^) compared with KaiC_SE_ in the presence (4.7 ± 0.3 h^–1^) and absence of KaiA_SE_ (0.08 ± 0.04 h^–1^) measured at 30 °C. Representative traces are shown, and the fitted parameters (mean ± s.d.) were obtained from three replicate measurements. **d**, X-ray structure of dodecameric KaiC_RS_ (PDB: 8DBA) coloured by hexamer A (light green) and hexamer B (dark green). The CI, CII and coiled-coil domains are indicated, and the A loop is labelled in **e**. **e**, Superposition based on an alignment of the CII domain of KaiC_RS_ (green; PDB: 8DBA, chain B), KaiC_SE_ (purple; PDB: 1TF7, chain B)^36^ and KaiC_SE_-S431E/T432A (yellow; PDB: 7S65, chain A)^19^ shows that KaiC_RS_ has an extended A loop orientation that no longer forms the inhibitory interaction with the 422 loop (KaiC_SE_ numbering). The conformation of the 422 loop in KaiC_RS_ resembles the one seen in the cryo-EM structure of the phosphomimetic KaiC_SE_-S431E/T432A (yellow; PDB: 7S65)^19^. No electron density is observed for the C-terminal part of wild-type KaiC_SE_ and the S431E/T432A mutant owing to flexibility, and the missing 22 residues for wild-type KaiC_SE_ (46 for S431E/T432A) are represented by a dashed line (not shown for the mutant).

**Extended Data Fig. 1:**
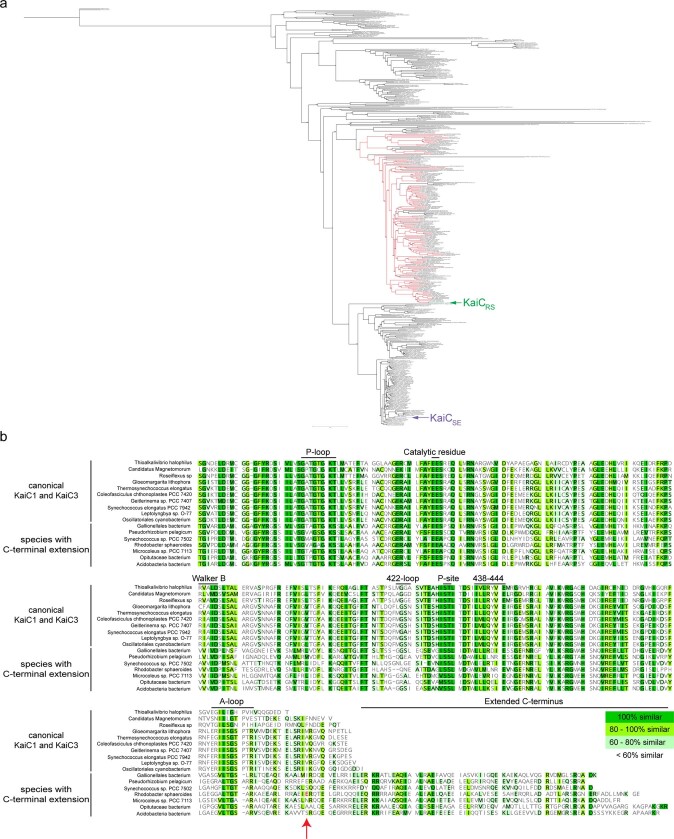
Evolution of *kaiC* and sequence alignment of *kaiC* subgroups. **(a)** Phylogenetic tree of *kaiC* homologs, where *kaiC* genes that have an approximately 50 amino acids C-terminal extension are labeled in red. *Rhodobacter sphaeroides* strain KD131 studied here and *Synechococcus elongatus* PCC 7942 (widely studied in the literature) are highlighted in green and pink, respectively. The accession code and organism are shown at the tip of the branches, the numbers at each node represent the aBayes bootstrap values^48^, and the legend for branch length is shown (see also Supplementary Datasets 1 and 2). **(b)** A sequence alignment of the CII domain of the *kaiC* subgroups annotated with its sequence similarity. Residue ﻿Glu490, the position where the stop codon was introduced in the truncated KaiC_RS_-Δcoil construct, is shown in red and marked with an arrow.

To gain insight into the evolution of the *kaiBC* cluster, we constructed a phylogenetic tree of *kaiC* after the emergence of *kaiB* (Fig. 1a, Extended Data Fig. 1a and Supplementary Datasets 1 and 2). The first question we sought to answer is how KaiC_RS_ and other members in the clade can autophosphorylate despite having no KaiA. KaiA is known to be crucial for this function in the canonical KaiABC system at its optimum temperature. We observed a large clade that exhibits a C-terminal tail about 50 amino acids longer compared with *kaiC* in other clades (Extended Data Fig. 1b). This C-terminal extension near the A loop is predominantly found in the *kaiC2* subgroup, which was previously annotated as having two serine phosphorylation sites instead of the Thr–Ser pair found in the *kaiC1* and *kaiC3* subgroups^13–15^ (Extended Data Fig. 1b). In *S.* *elongatus*, the binding of KaiA_SE_ to the A loop of KaiC_SE_ tethers them in an exposed conformation^16^ that activates both autophosphorylation and nucleotide exchange^17^. Given the proximity of the extended C-terminal tail to the A loop, we conjectured that it could serve as the ‘primitive’ regulatory moiety that was made redundant with the appearance of KaiA.

**Extended Data Fig. 2:**
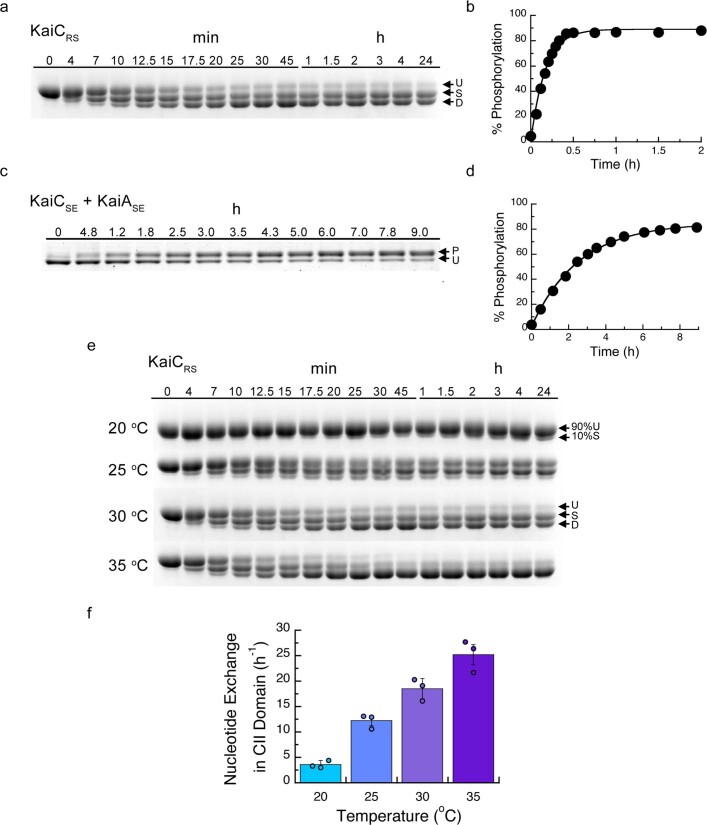
Auto-phosphorylation and nucleotide exchange rates of KaiC. **(a)** 10% SDS-PAGE gel of 3.5 μM KaiC_RS_ in the presence of 4 mM ATP and using an ATP-recycling system at 30 °C. U, S, and D represent unphosphorylated, singly, and doubly phosphorylated KaiC_RS_, respectively. **(b)** Densitometric analysis of auto-phosphorylation (single + double phosphorylation) from panel (a) over time yields a rate of 6.5 ± 1.0 h^−1^. **(c)** 6.5% SDS-PAGE gel of 3.5 μM KaiC_SE_ in the presence of 1.2 μM KaiA_SE_ and 4 mM ATP at 30 °C. U and P represent unphosphorylated and phosphorylated KaiC_SE_, respectively. **(d)** Densitometric analysis of auto-phosphorylation of KaiC_SE_ activated by KaiA_SE_ (panel (c)) shows a rate of 0.40 ± 0.02 h^−1^ and is substantially slower than for KaiC_RS_. The standard deviation for parameters in (b) and (d) were obtained from data fitting. **(e)** 10% SDS-PAGE gels for experiments with 3.5 μM KaiC_RS_ in the presence of 4 mM ATP and using an ATP-recycling system between 20 and 35 °C show that the level of phosphorylation increases with temperature. U, S, and D represent unphosphorylated, singly, and doubly phosphorylated KaiC_RS_, respectively. For gel source data in (a), (c), and (e), see Supplementary Figure 2. **(f)** Bar graphs indicating the nucleotide exchange rate in the CII domain of KaiC_RS_ incubated with 50 μM ATP in the presence of an ATP-recycling system, and then mixed with 250 μM mant-ATP. An increase in fluorescence intensity at 440 nm was recorded and the single-exponential time traces were fitted to obtain the exchange rate constants: 3.6 ± 0.8 h^−1^ (20 °C), 12.2 ± 1.0 h^−1^ (25 °C), 18.5 ± 1.5 h^−1^ (30 °C), and 25.2 ± 0.2 h^−1^ (35 °C). Experiments were performed in triplicate and data are presented as mean values ± s.d.

To test our hypothesis, we first measured the autophosphorylation and nucleotide exchange rates in KaiC_RS_, which both depend on the presence of KaiA in the KaiABC_SE_ system. We observed an autophosphorylation rate for KaiC_RS_ that was about 16-fold higher than for KaiC_SE_ activated by KaiA_SE_ (6.5 ± 1.0 h^−1^ compared with 0.40 ± 0.02 h^−1^, respectively; Fig. 1b and Extended Data Fig. 2a–e). Similarly, the nucleotide exchange rate was faster in KaiC_RS_ compared with KaiC_SE_, even in the presence of KaiA_SE_ (18.0 ± 1.5 h^−1^ compared with 4.7 ± 0.3 h^−1^, respectively; Fig. 1c and Extended Data Fig. 2f). Our data show that KaiC_RS_ can perform both autophosphorylation and nucleotide exchange on its own and does so faster than its more recently evolved counterparts.

## A coiled-coil interaction assembles a KaiC_RS_ dodecamer

To mechanistically assess how KaiC in *kaiA*-null systems accomplishes autophosphorylation, we turned to structural biology. The crystal structure of KaiC_RS_, unlike KaiC from cyanobacteria, revealed a homododecamer that consisted of two homohexameric domains joined by a 12-helical coiled-coil domain that is formed by the extended C-terminal tail (Protein Data Bank (PDB) identifier: 8DBA; Fig. 1d and Extended Data Table 1). A closer inspection of the CII domains in KaiC_RS_ and KaiC_SE/TE_ (*Thermosynechococcus elongatus* BP-1 referred to as KaiC_TE_) showed an obvious difference in A loop orientations: an extended conformation in KaiC_RS_ compared with a buried orientation in KaiC_SE/TE_ (Fig. 1e). The existence of such an extended conformation following binding of KaiA has been previously proposed^18^. This hypothesis was based on the perceived hyperphosphorylation and hypophosphorylation that occurred after removing the A loop or disrupting KaiA binding, respectively^18^. A recently solved cryo-EM structure of the night-time phosphomimetic KaiC_SE_-S431E/T432A in its compressed state directly showed a disordered A loop that no longer interacts with the 422 loop^19^, similar to the extended A loop conformation we observed in KaiC_RS_ (Fig. 1e). The loss of interaction between the A loop and the 422 loop (just 10 residues apart from the phosphorylation sites) results in closer proximity between the hydroxyl group of Ser431–Thr432 and the γ-phosphate of ATP, thereby, facilitating the phosphoryl transfer step^20^. Furthermore, the sequence similarity between KaiC_RS_ and KaiC_SE_ is less than 30% for the A loop and residues considered important for stabilization of this loop in its buried orientation (that is, the 422 loop and residues 438–444) (Fig. 1e). Together, our structural and kinetic data support the idea that an exposed A loop is key for the KaiA-independent enhancement of nucleotide exchange and hence autophosphorylation in KaiC_RS_ and perhaps other KaiBC-based systems.

**Fig. 2:**
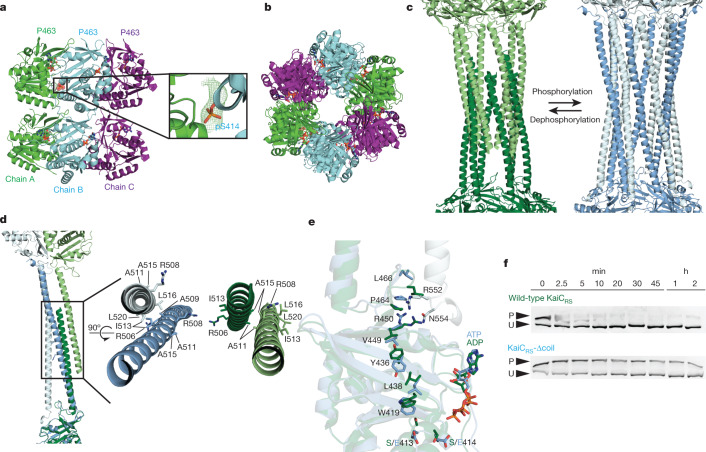
A coiled-coil partner switch coupled to an allosteric network in the CII domain promotes autodephosphorylation. **a**, X-ray structure of KaiC_RS_-Δcoil was solved in the C222_1_ space group and contained three monomers in the asymmetric unit, with ADP present in all active sites. The obtained electron density map allowed for model building up to Pro463, which indicated that the truncation at position 490 results in enhanced flexibility beyond Pro463. Phosphorylation of Ser414 (pS414) was observed in chain B (cyan) as shown by the electron density *mF*_o_–*DF*_c_ polder map (green mesh, 3*σ* contour level). **b**, Assembly analysis using the PISA software^37^ revealed a hexamer as the most probable quaternary structure (top view). **c**, Structural comparison of the coiled-coil domain for unphosphorylated KaiC_RS_ (dark and light green; X-ray structure) and the KaiC_RS_-S413E/S414E phosphomimetic mutant (dark and light blue; cryo-EM structure). **d**, Overlay of interacting dimers of the structures in **c** using the CII domain of chain A as a reference (dark shades; bottom). Unphosphorylated KaiC_RS_ (dark green) interacts with the opposite partner on the right (light green), whereas KaiC_RS_-S413E/S414E (dark blue) interacts with the partner on the left (light blue). The hydrophobic packing in the coiled-coil domain is mediated by only the Cβ atoms of alanine and arginine residues in unphosphorylated KaiC_RS_, but involves the entire side chain of leucine and isoleucine residues in the phosphomimetic structure. **e**, Allosteric network in the phosphomimetic state (blue) from the coil (light blue) propagating through the KaiC_RS_ CII domain to the active site (dark blue) compared with the unphosphorylated state (dark green) (Supplementary Video 1). **f**, Autodephosphorylation of KaiC_RS_ and KaiC_RS_-Δcoil over time in the presence of 4 mM ADP at 30 °C. The phosphorylated (P) and unphosphorylated (U) proteins were separated by Zn^2+^ Phos-tag SDS–PAGE (for gel source data, see Supplementary Fig. 1).

**Extended Data Fig. 3:**
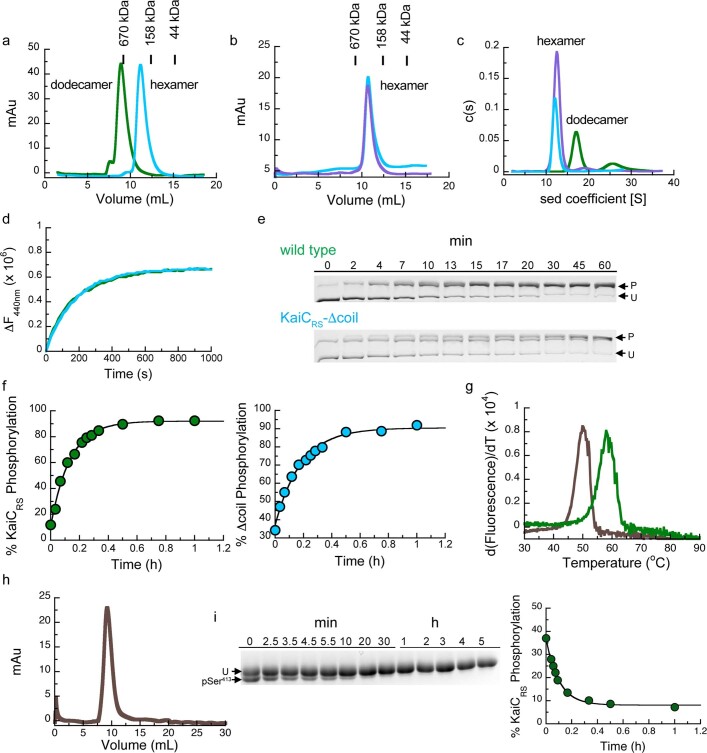
Oligomeric states of KaiC_RS_ and effect of coiled-coil domain on rates of nucleotide exchange and auto-phosphorylation. **(a)** Oligomerization analysis of KaiC_RS_ (dodecamer, green line) and truncated KaiC_RS_-Δcoil (hexamer, cyan line) by analytical gel-filtration chromatography. The protein size markers are indicated at the top. **(b)** Comparison of the elution profiles of KaiC_RS_-Δcoil (cyan line) and KaiC_SE_ (purple line) from size-exclusion chromatography shows a hexameric state for both KaiC_RS_-Δcoil and KaiC_SE_. **(c)** Oligomeric states of KaiC_RS_ (dodecamer, green line), KaiC_RS_-Δcoil (hexamer, cyan line), and KaiC_SE_ (hexamer, purple line) were also measured by analytical ultracentrifugation (sedimentation velocity at 30,000 rpm and 20 °C) and the results agree with the data shown in panels (a) and (b). The graph in panel (c) represents the sedimentation coefficient distribution [c(s)]. **(d)** The change in fluorescence at 440 nm (ΔF_440nm_) represents the nucleotide exchange between ATP and mant-ATP at 30 °C for KaiC_RS_ (green trace, 18.0 ± 1.5 h^−1^) and KaiC_RS_-Δcoil (cyan trace, 19.1 ± 0.8 h^−1^). Representative traces are shown and the fitted parameters (mean ± s.d.) were obtained from three replicate measurements. **(e)** Zn^2+^ Phos-tag^TM^ SDS-PAGE gel shows the level of phosphorylation over time of KaiC_RS_ (upper gel) and KaiC_RS_-Δcoil (lower gel) at 35 °C. P and U represent phosphorylated and unphosphorylated protein, respectively. **(f)** Phosphorylation level over time of KaiC_RS_ (green circles, 7.4 ± 0.3 h^−1^) and KaiC_RS_-Δcoil (cyan circles, 5.5 ± 0.4 h^−1^) analyzed by densitometric analysis of Zn^2+^ Phos-tag^TM^ SDS-PAGE gel in (e). **(g)** First derivative of thermal-stability curves measured for unphosphorylated KaiC_RS_ bound with ADP (brown line) and phosphorylated KaiC_RS_ bound with ATP (green line). The extracted temperatures of denaturation are 50 °C (unphosphorylated KaiC_RS_ in the presence of 1 mM ADP) and 58 °C (phosphorylated KaiC_RS_ in the presence of 1 mM ATP), respectively. **(h)** Dodecameric state of unphosphorylated KaiC_RS_ (40 μM) bound with ADP measured by size-exclusion chromatography. **(i)** SDS-PAGE gel shows dephosphorylation of Ser413 over time at 30 °C in the presence of 4 mM ADP (U and pSer413 represent unphosphorylated and Ser413-phosphorylated KaiC_RS_, respectively) with the corresponding kinetics shown in the right panel (confirmed by MS/MS) with a rate constant of 11.5 ± 0.8 h^−1^. This result suggests that the coiled-coil domain promotes KaiC_RS_ dephosphorylation. For gel source data in (e) and (i), see Supplementary Figure 2. The standard deviation for parameters in (f) and (i) were obtained from data fitting.

We then questioned whether the purpose of the coiled-coil domain is to ‘pull up’ the A loop or to actively participate in nucleotide exchange and autophosphorylation of KaiC. To further understand its role, we generated a truncation at residue Glu490 based on the phylogenetic tree and crystallographic information (KaiC_RS_-Δcoil) (Extended Data Fig. 1b) to disrupt the coiled-coil interaction between the two hexamers. The crystal structure of KaiC_RS_-Δcoil (PDB: 8DB3; Fig. 2a,b and Extended Data Table 1), its size-exclusion chromatogram and analytical ultracentrifugation profile (Extended Data Fig. 3a–c) showed a hexameric structure with no coiled-coil interaction. Nucleotide exchange rates in the CII domain for KaiC_RS_-Δcoil and the wild-type protein were comparable (19.1 ± 0.8 h^−1^ and 18.0 ± 1.5 h^−1^, respectively; Extended Data Fig. 3d). The phosphorylation rates were also similar (5.5 ± 0.4 h^−1^ and 7.4 ± 0.3 h^−1^ for KaiC_RS_-Δcoil and wild type, respectively; Extended Data Fig. 3e,f). These results indicate that the extended A loop and not the coiled-coil interaction plays a pivotal part in nucleotide exchange and autophosphorylation in KaiC_RS_. The results also provide a potential mechanism of autophosphorylation in other KaiBC-based systems that lack a coiled-coil bundle. Notably, the coiled-coil bundle provides additional hexameric stability. In detail, the KaiC_RS_ dodecamer is stable for extended periods of time in the presence of only ADP (Extended Data Fig. 3g,h), whereas for KaiC_SE_, oligomers are not observed under these conditions^21^.

## A long-range allosteric network in KaiC_RS_

**Extended Data Fig. 4:**
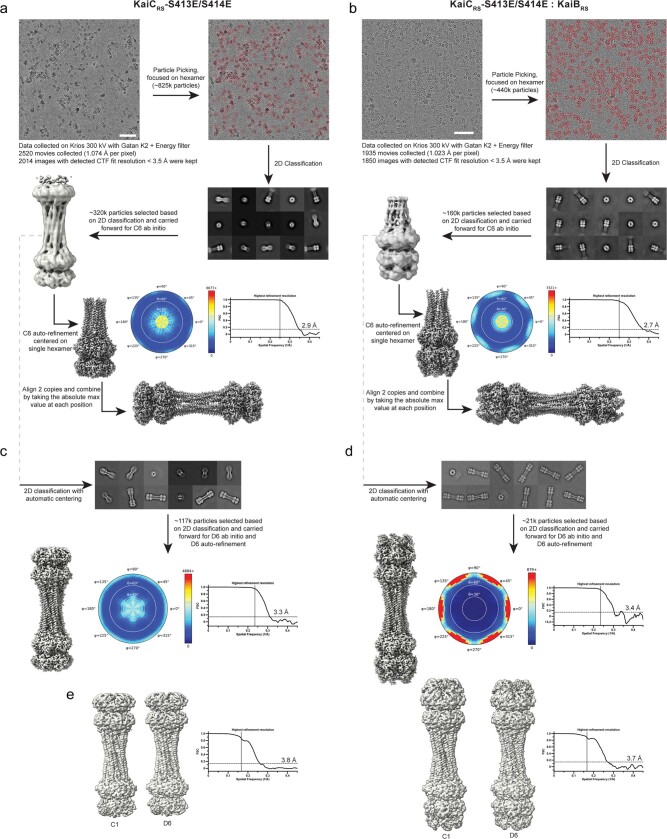
Graphical description of the cryo-EM processing workflow and validation of the final dodecamer structures. The workflow (see description in Methods section) demonstrates a typical image (scale bar: 60 nm) and representative good class averages. The ab initio and final reconstructions are shown. Shown alongside the final reconstruction is the angular plot demonstrating the distribution of particle views and the Fourier shell correlation curve used for the global resolution estimation **(a)** KaiC_RS_-S413E/S414E alone and **(b)** KaiC_RS_-S413E/S414E:KaiB_RS_ complex. To validate the final combined dodecamer structures, the data were reprocessed for the full dodecamer. The figure shows representative good class averages, the final reconstruction, angular distribution and Fourier shell correlation curve for the **(c)** KaiC_RS_-S413E/S414E alone and **(d)** KaiC_RS_-S413E/S414E:KaiB_RS_ complex dodecamers. **(e)** Comparison of the *C*1 and *D*6 reconstructions of KaiC_RS_-S413E/S414E alone and KaiC_RS_-S413E/S414E:KaiB_RS_, and Fourier shell correlation curves for the *C*1 reconstructions. The *C*1/*D*6 comparisons do not reveal discernable differences, suggesting that these complexes have *D*6 symmetry.

**Extended Data Fig. 5:**
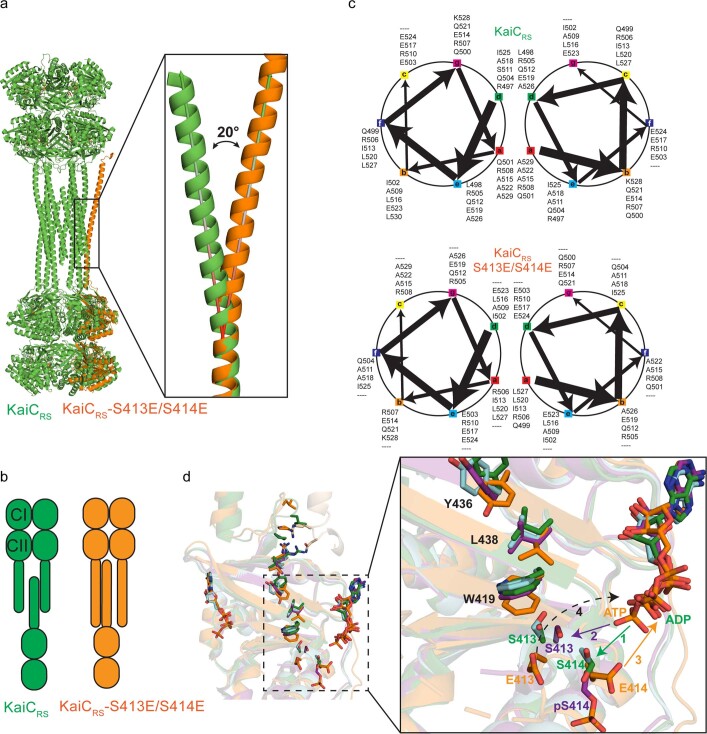
Correlation between the coiled-coil register shift and phosphorylation, and model for consecutive phosphorylation/dephosphorylation events in CII domain of KaiC_RS_. **(a)** Structural comparison between KaiC_RS_ (green) and KaiC_RS_-S413E/S414E (orange, single-chain for clarity) reveals that the coiled-coil in the phosphomimetic structure points outwards, with an angle of about 20° relative to the KaiC_RS_ coiled-coil. **(b)** The conformational change in the coiled-coil domain affects the dimer interface due to partner swaps with the opposite hexamer (see also Fig. 2). From an “outside perspective”: the C-terminal helix in KaiC_RS_ interacts with the right chain from the opposite hexamer, whereas in KaiC_RS_-S413E/S414E the interaction is with the chain on the left. **(c)** Coiled-coil diagrams describe the heptad register shift that accompanies this structural rearrangement. **(d)** Based on the overlay of our structures, we propose the following model for the phosphorylation/dephosphorylation events. First, the phosphorylation cycle starts with the transfer of the γ-phosphate of ATP to the hydroxyl group of Ser414 (1; green arrow) in unphosphorylated KaiC_RS_ (green) or KaiC_RS_-Δcoil (cyan). Secondly, pSer414 of KaiC_RS_-Δcoil (purple, singly phosphorylated) moves away from the active site placing the hydroxyl group of Ser413 closer to the γ-phosphate of ATP for the second phosphorylation (2; purple arrow). Thirdly, the doubly phosphomimetic state (KaiC_RS_-S413E/S414E, orange) reveals that the phosphoryl group of pSer414 moves back towards the active site for dephosphorylation (3; orange arrow). Lastly, we hypothesize that the indole group of Trp419 “pushes” pSer413 into the active site for the second dephosphorylation event (dashed arrow), in agreement with the slower dephosphorylation rate observed in the KaiC_RS_-Δcoil construct (*cf*. Fig. 2d).

The change in phosphorylation state of KaiC has been well established to be the central feature for the circadian rhythm^22,23^. Notably, when comparing the unphosphorylated form of full-length KaiC_RS_ (PDB: 8DBA) and its phosphomimetic mutant (S413E/S414E; PDB: 8FWI) (Extended Data Fig. 4 and Extended Data Table 2), we observed two distinct coiled-coil interactions. Following phosphorylation, the coiled-coil pairs swap partners by interacting with the other neighbouring chain from the opposite hexamer, which resulted in a register shift that propagated around 115 Å along the entire coiled-coil (Fig. 2c and Extended Data Fig. 5). In the phosphomimetic state, the register comprised bulkier hydrophobic residues that resulted in a more stable interaction than for the dephosphorylated form (Fig. 2d and Extended Data Fig. 3g). Furthermore, the C-terminal residues of KaiC_RS_-S413E/S414E interacted with the CII domain of the opposite hexamer, whereas the lack of electron density for the last 30 residues in the wild-type structure indicates more flexibility in the dephosphorylated state. We discovered that these conformational changes in the coiled-coil domain seemed to be coupled through a long-range allosteric network to the phosphorylation sites. The rotameric states of residues Ser413, Ser414, Trp419, Val421, Tyr436, Leu438, Val449 and Arg450 moved concertedly and pointed towards the nucleotide-binding site when the protein was phosphorylated or pointed away in the absence of a phosphate group (Fig. 2e, Extended Data Fig. 5d and Supplementary Video 1). We propose that the proximity of the nucleotide to the phosphorylated residue facilitated more efficient phosphoryl transfer. We therefore experimentally determined the impact of the coiled-coil domain on the autodephosphorylation rate of KaiC_RS_. The wild-type protein dephosphorylated comparatively quickly (observed rate constant = 11.5 ± 0.8 h^−1^) in the presence of only ADP. By contrast, little dephosphorylation was observed for KaiC_RS_-Δcoil (Fig. 2f and Extended Data Fig. 3i), for which allosteric propagation was disrupted (Extended Data Fig. 5d). Consistent with this accelerated dephosphorylation rate mediated by the coiled-coil domain, our crystallographic data showed a phosphate group on Ser414 for KaiC_RS_-Δcoil but not for the wild-type protein (Fig. 2a and Extended Data Fig. 5d).

## The ATP-to-ADP ratio resets the clock

**Fig. 3:**
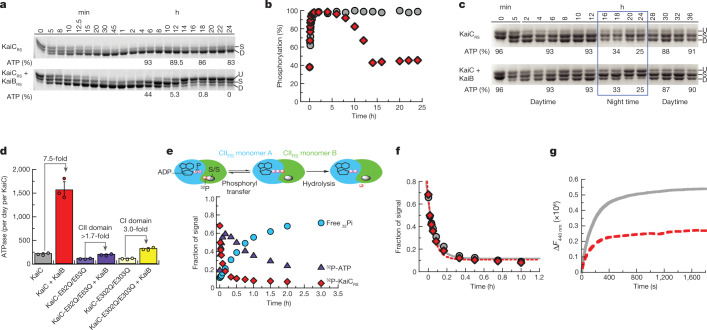
The regulatory role of KaiB_RS_ in the phosphorylation–dephosphorylation cycle of KaiC_RS_. **a**, SDS–PAGE gel of 3.5 μM KaiC_RS_ and 4 mM ATP in the absence (top) and presence (bottom) of 3.5 μM KaiB_RS_ at 35 °C, with the percentage of ATP indicated at specific time points. **b**, Phosphorylation (single and double) of KaiC_RS_ during the reaction in the absence (grey circles) or presence (red diamonds) of KaiB_RS._ **c**, Phosphorylation–dephosphorylation cycle of 3.5 μM phosphorylated KaiC_RS_ in the absence and presence of 3.5 μM KaiB_RS_ in a constant ATP-to-ADP ratio of high ATP (4 mM) to mimic daytime and about 25% ATP to mimic the night time (exact percentage of ATP indicated at specific time points) at 30 °C. U, S and D in **a** and **c** represent the unphosphorylated, single phosphorylated (at Ser413 or Ser414) and double phosphorylated state of KaiC_RS_, respectively (for gel source data, see Supplementary Fig. 1). **d**, ATPase activity of wild-type KaiC_RS_ in the absence and presence of KaiB_RS_, KaiC_RS_-E62Q/E63Q in the absence and presence of KaiB_RS_, and KaiC_RS_-E302Q/E303Q in the absence and presence of KaiB_RS_ at 30 °C. Bar graphs show mean ± s.d. from three replicates. **e**, Time-dependent autodephosphorylation of ^32^P-labelled KaiC_RS_ bound with ADP in the presence of 20 μM KaiB_RS_ and 4 mM ADP at 30 °C showing phosphorylated ^32^P-KaiC_RS_, ^32^P-ATP and free ^32^Pi. The reaction products were separated by thin layer chromatography. **f**, The decay of phosphorylated ^32^P-KaiC_RS_ bound with 4 mM ADP in the absence (grey circles) and presence (red diamonds) of KaiB_RS_ at 30 °C is obtained from autoradiography quantification (Extended Data Fig. 7). **g**, The nucleotide exchange of 3.5 μM KaiC_RS_ (grey trace) and 3.5 μM KaiC_RS_ in complex with 30 μM KaiB_RS_ (red dotted trace) in the presence of ATP with mant-ATP. Representative traces are shown, and the fitted parameters (mean ± s.d.) were obtained from three replicate measurements.

**Extended Data Fig. 6:**
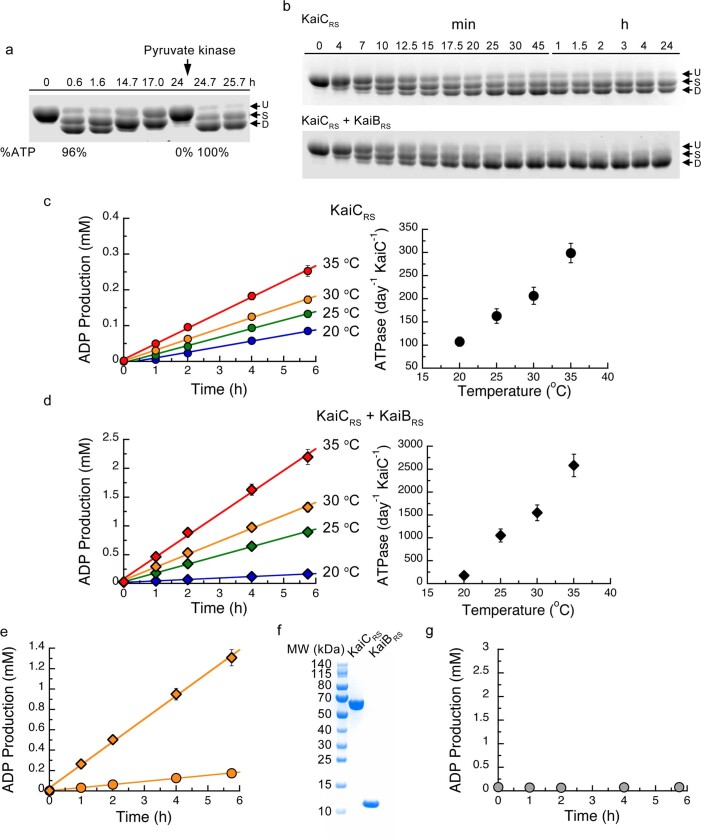
Effect of ATP-to-ADP ratio on KaiC_RS_ auto-dephosphorylation and the dependence of temperature and KaiB_RS_ binding on the ATPase activity of KaiC_RS_. **(a)** 10% SDS-PAGE gel of 3.5 μM KaiC_RS_ and 3.5 μM KaiB_RS_ in the presence of 4 mM ATP and 10 mM 2-phosphoenolpyruvate at 30 °C shows that the auto-phosphorylation cycle restarts upon regeneration of ATP by the addition of 2 U ml^−1^ pyruvate kinase at the 24-hour time mark. **(b)** 10% SDS-PAGE gel of 3.5 μM KaiC_RS_ without (upper panel) and with (lower panel) 3.5 μM KaiB_RS_ in the presence of 4 mM ATP with an ATP-recycling system added from the beginning showing that under these conditions KaiB does not accelerate dephosphorylation. For gel source data in (a) and (b), see Supplementary Figure 2. **(c)** Representative curves for ADP production of KaiC_RS_ (3.5 μM) alone and **(d)** in the presence of KaiB_RS_ (3.5 μM) in 4 mM ATP measured by HPLC. The data were analysed as described in the Methods section and result in ATPase activities of 108 ± 10 day^−1^ KaiC^−1^ (with KaiB_RS_ = 176 ± 29 day^−1^ KaiC^−1^) at 20 °C, 163 ± 16 day^−1^ KaiC^−1^ (with KaiB_RS_ = 1052 ± 143 day^−1^ KaiC^−1^) at 25 °C, 208 ± 19 day^−1^ KaiC^−1^ (with KaiB_RS_ = 1557 ± 172 day^−1^ KaiC^−1^) at 30 °C, and 300 ± 21 day^−1^ KaiC^−1^ (with KaiB_RS_ = 2584 ± 245 day^−1^ KaiC^−1^) at 35 °C. The temperature coefficient, *Q*_10_, was calculated using the data obtained at 25 °C and 35 °C and yields a value of ~ 1.9. The standard deviations of ATPase activity at each temperature (right panels) were obtained from three replicate measurements and data are presented as mean values ± s.d. **(e)** The comparison of ADP production of KaiC_RS_ in the absence (orange circles, data from panel (c)) and presence (orange diamonds, data from panel (d)) of KaiB_RS_ at 30 °C indicate a 7.5-fold increase in ATPase activity for the complex. The binding of KaiB_RS_ accelerates the ATPase activity of KaiC_RS_ in both the CI and CII domains (see also Extended Data Fig. 8b, c). **(f)** The SDS-PAGE gel of KaiC_RS_ (10 μg) and KaiB_RS_ (10 μg) shows that both proteins were purified to homogeneity and the measured ATPase activity is, therefore, not due to impurities. **(g)** ADP production of KaiB_RS_ in 4 mM ATP at 30 °C shows, as expected, no ATPase activity for KaiB_RS_ alone and confirms the increase in ATPase activity shown in panel (d) is due to complex formation. The standard deviation for the representative curves shown in panels (c-d, left), e, and g was set to 6% assuming the largest systematic error originates from the injector.

It was notable that KaiC_RS_ can autodephosphorylate on its own despite being constitutively active for phosphorylation owing to its extended A loop conformation. In the canonical *kaiABC* system, the interaction between KaiB and KaiC is required to provide a new binding interface that sequesters KaiA from its activating binding site, thereby promoting autodephosphorylation at the optimum temperature of the organism^24–26^. We therefore sought to discover whether the KaiC_RS_ system can oscillate and whether there is a regulatory role for KaiB_RS_ in this process. Comparing the in vitro phosphorylation states of KaiC_RS_ in the absence and presence of KaiB_RS_ showed an initial, rapid phosphorylation followed by an oscillatory-like pattern in the presence of KaiB_RS_ (hereafter referred to as KaiBC_RS_), whereas KaiC_RS_ alone remained phosphorylated (Fig. 3a,b). Notably, the ATP consumption during the reaction with KaiB_RS_ was significantly higher than without (Fig. 3a). As noted above, KaiC_RS_ will also dephosphorylate completely in the presence of only ADP (Fig. 2f). These results suggest that the phosphorylation state of KaiC_RS_ and the observed oscillatory half-cycle (Fig. 3a,b) is probably related to a change in the ATP-to-ADP ratio. We conjectured that this could constitute the environmental cue to reset the timer. To test our hypothesis, an ATP-recycling system was added after complete dephosphorylation of KaiBC_RS_. As predicted, KaiC_RS_ was able to restart the cycle and phosphorylate again (Extended Data Fig. 6a). We note that in vivo, the ATP-to-ADP ratio will not vary as substantially as in this in vitro experiment, as nucleotide homeostasis is tightly regulated. To mimic the day and night period for *R.* *sphaeroides*, we repeated the experiments while keeping the ATP-to-ADP ratio constant (mostly ATP at daytime owing to photosynthesis compared with 25:75% ATP-to-ADP during night time)^27^. In the presence of high ATP (that is, mimicking daytime), KaiC_RS_ remained single or double phosphorylated (Fig. 3c and Extended Data Fig. 6b) irrespective of KaiB_RS_. By contrast, a constant 25:75% ATP-to-ADP ratio (that is, mimicking night time) resulted in a much higher fraction of dephosphorylated KaiC_RS_ in the presence of KaiB_RS_ (Fig. 3c). Moreover, when the ATP-to-ADP ratio was flipped to mimic daytime, KaiC_RS_ was able to phosphorylate again (Fig. 3c, around the 28 h mark). Our data support the notion that the phosphorylation behaviour of KaiBC_RS_ strongly depends on the ATP-to-ADP ratio and demonstrate that the physical binding of KaiB_RS_ results in a higher level of KaiC_RS_ dephosphorylation at night time.

Next we investigated the accelerated ATPase activity observed in KaiC_RS_ after the formation of the complex. The ATPase activity reported for KaiC_SE_ is low (about 15 ATP molecules per day per molecule of KaiC_SE_) and was proposed as a reason for the slowness of circadian oscillation^28^. KaiC_RS_ alone shows a significantly faster ATPase rate than KaiC_SE_, which is further enhanced by binding of KaiB_RS_ (208 ± 19 and 1,557 ± 172 ATP molecules day per day per KaiC_RS_, respectively; left two bars in Fig. 3d and Extended Data Fig. 6c–g). Furthermore, KaiC_RS_ does not exhibit temperature compensation for its ATPase activity (temperature coefficient *Q*_10_ about 1.9; Extended Data Fig. 6c), a feature that is present in KaiC_SE_ and proposed to be a prerequisite for self-sustained rhythms^28^. The deviation from unity for *Q*_10_ is consistent with our earlier observation that the KaiBC_RS_ system is not a true circadian oscillator but rather an hourglass timer (Fig. 3b).

## Regulatory role of KaiB_RS_

**Extended Data Fig. 7:**
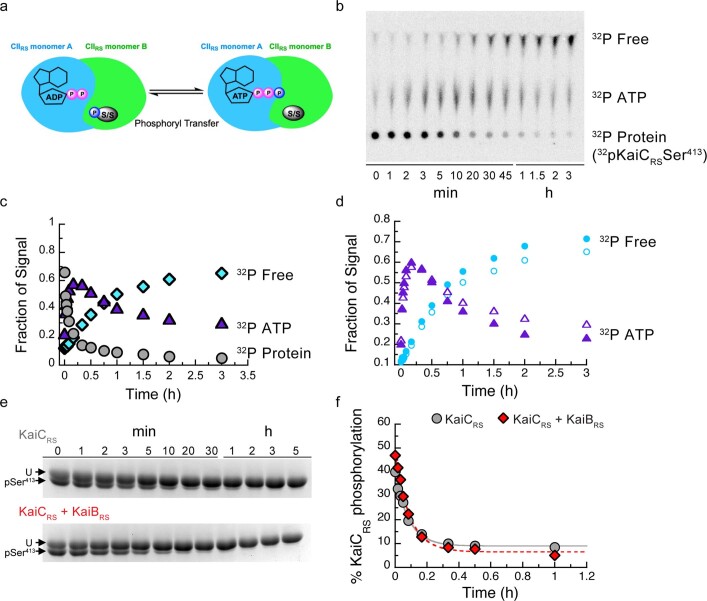
Dephosphorylation of KaiC_RS_ occurs via an ATP-synthase mechanism and the phosphoryl-transfer step is unaffected by the binding of KaiB_RS_. **(a)** Possible mechanisms how KaiB_RS_ could accelerate KaiC_RS_ dephosphorylation at nighttime. Binding of KaiB_RS_ on the CI_RS_ domain directly accelerates the phosphoryl transfer from pSer to bound ADP to generate transiently bound ATP. The cartoon represents the interface between two monomers in the CII_RS_ domain. **(b)** Autoradiograph of separation of ^32^P-KaiC_RS_ at Ser413, transiently formed ^32^P-ATP, and free ^32^Pi via thin-layer chromatography (TLC) with 4 mM ADP at 30 °C, with the corresponding kinetics shown in **(c)** where gray circle, purple triangle, and cyan diamonds represent the relative concentrations of phosphorylated ^32^P-KaiC_RS_, ^32^P-ATP, and free ^32^Pi, respectively. **(d)** Comparison of transient ^32^P-ATP formation and decay in the absence (open triangle) and presence (solid triangle) of KaiB_RS_ and free ^32^P formation in the absence (open circles) and presence of KaiB_RS_ (solid circles). Faster decay of transient ^32^P-ATP together with higher free ^32^P production in the presence of KaiB_RS_ indicated that KaiB_RS_ accelerates hydrolysis in KaiC_RS_. **(e)** SDS-PAGE gel (10%) of dephosphorylation of phosphorylated 3.5 μM KaiC_RS_ at Ser413 without (upper gel) and with (lower gel) 3.5 μM KaiB_RS_ in the presence of 4 mM ADP at 30 °C (for gel source data, see Supplementary Figure 2). **(f)** Densitometric analysis of data in panel (e) shows the decay of total KaiC_RS_ phosphorylation in the absence (gray circles) and presence (red diamonds) of KaiB_RS_ and yields rates of 11.5 ± 0.8 h^−1^ and 11.0 ± 0.8 h^−1^, respectively. This result indicates that binding of KaiB_RS_ does not accelerate the phosphoryl-transfer step in KaiC_RS_.

**Extended Data Fig. 8:**
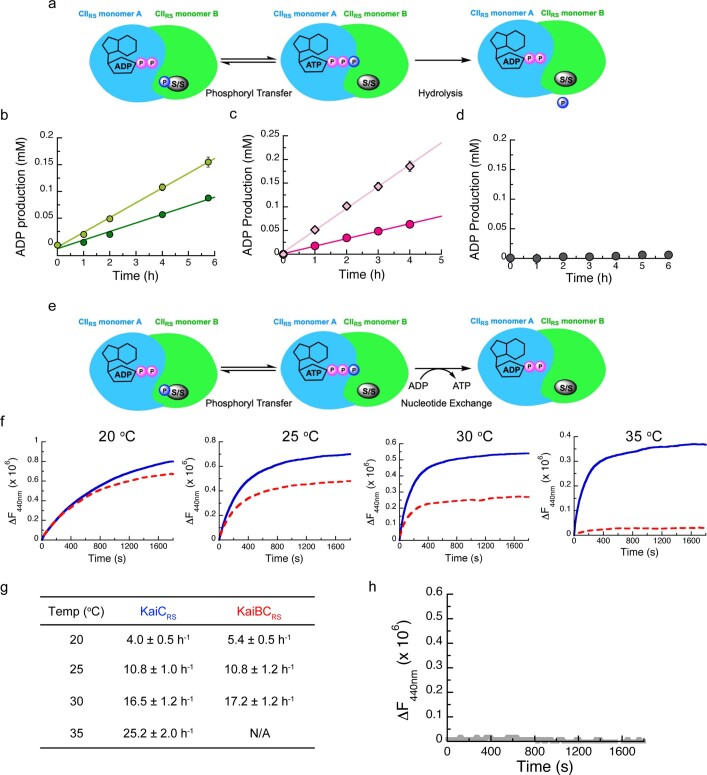
Effect of KaiB_RS_ binding on ATPase activity and nucleotide exchange in the CII domain of KaiC_RS_. **(a)** Second possible mechanism to explain how KaiB_RS_ accelerates KaiC_RS_ dephosphorylation at nighttime**:** binding of KaiB_RS_ to the CI_RS_ domain could increase the hydrolysis rate in the CII_RS_ domain and, thereby, prevent the phosphoryl transfer back from transiently formed or external ATP back to serine residues. **(b)** Representative curves for ADP production of phosphorylated KaiC_RS_ with catalytic mutations in the CI domain (KaiC_RS_-E62Q/E63Q, 3.5 μM) in the absence (dark green circles) and presence (light green circles) of 3.5 μM KaiB_RS_ at 30 °C with 4 mM ATP was quantified using HPLC. From these data an ATPase activity in the CII domain of 112 ± 8 day^−1^ KaiC^−1^ and 195 ± 16 day^−1^ KaiC^−1^ in the presence of KaiB_RS_ was determined. **(c)** Representative curves for ADP production measured by HPLC as in panel (b) of KaiC_RS_ but with catalytic mutations in the CII domain (KaiC_RS_-E302Q/E303Q, 3.5 μM) in the absence (dark pink circles) and presence (light pink diamonds) of 3.5 μM KaiB_RS_. The corresponding ATPase activities in the CI domain are 110 ± 12 day^−1^ KaiC^−1^ in the absence and 320 ± 22 day^−1^ KaiC^−1^ in the presence of KaiB_RS_. **(d)** Representative curves for ADP production of KaiCI_RS_-E62Q/E63Q (construct of only CI domain with catalytic mutations) in 4 mM ATP at 30 °C shows no ATPase activity indicating that Glu62 and Glu63 are the only two residues that are responsible for ATPase activity in CI domain of KaiC_RS_ and confirms the ATPase activity shown in panel b is due to ATPase activity in CII domain of KaiC_RS_. The standard deviation for the representative curves shown in panels (b-d) was set to 6% assuming the largest systematic error originates from the injector. The experiments in panel (b-d) were performed in triplicate and ATPase rate given in the legend for panels (b) and (c) are presented as mean values ± s.d. **(e)** Third possible mechanism to explain how KaiB_RS_ accelerates KaiC_RS_ dephosphorylation at nighttime: binding of KaiB_RS_ to the CI_RS_ domain could promote faster nucleotide exchange in the CII_RS_ domain to displace transient ATP by ADP. **(f)** Time course of fluorescence intensity at 440 nm due to binding of mant-ATP to KaiC_RS_-S413E bound with ATP in the absence (solid blue trace) and presence (red dotted trace) of KaiB_RS_. KaiC_RS_-S413E (3.5 μM) was pre-incubated with 3.5 μM KaiB_RS_ for 16 h at 20, 25, 30, and 35 °C in the presence of 50 μM ATP and an ATP-recycling system and then mixed with 250 μM mant-ATP. The observed exchange rates at each temperature are listed in the table **(g)**. **(h)** Nucleotide exchange of KaiCI_RS_ (i.e., only CI_RS_ domain) cannot be measured since there is no tryptophan residue in close proximity of the nucleotide binding site. In summary, KaiB_RS_ accelerates KaiC_RS_ dephosphorylation by increasing the hydrolysis rate in the CI and CII domains and does not affect the nucleotide exchange rate. Representative traces are shown in (f) and (h) and the fitted parameters (g; mean ± s.d.) were obtained from three replicate measurements.

Mechanistic details of how the binding of KaiB_RS_ in the CI domain allosterically affects the autodephosphorylation of KaiC_RS_ in the CII domain remain unclear. There are three plausible scenarios to explain this: (1) KaiB_RS_ binding stimulates the phosphoryl transfer from pSer back to ADP (Extended Data Fig. 7a); (2) KaiB_RS_ binding increases the hydrolysis rate of the active-site ATP (Extended Data Fig. 8a); or (3) KaiB_RS_ binding accelerates nucleotide exchange in the CII domain (Extended Data Fig. 8e). To differentiate among these possibilities, we performed radioactivity experiments to follow nucleotide interconversion. We also measured ATPase activity for wild-type KaiC_RS_ and mutant forms that are incapable of ATPase activity in the CI or CII domain, and quantified nucleotide-exchange rates by measuring the fluorescence of mant-ATP. First, we detected fast, transient ^32^P-ATP formation in our radioactivity experiments when starting from ^32^P-phosphorylated KaiC_RS_, which was due to its ATP synthase activity in the CII domain (Fig. 3e and Extended Data Fig. 7b–d). The observed phosphoryl-transfer rate was independent of KaiB_RS_ (observed rate constant = 12.0 ± 1.7 h^−1^ and 15.4 ± 1.7 h^−1^ in its absence and presence, respectively; Fig. 3f) and agreed well with the rates determined from our gel electrophoresis experiments (11.0 ± 0.8 h^−1^ and 11.5 ± 0.8 h^−1^ with or without KaiB_RS_, respectively; Extended Data Fig. 7e,f). Our experimental data confirmed that KaiC_RS_ undergoes dephosphorylation through an ATP synthase mechanism, similar to what was observed for KaiC_SE_ (ref. ^29^). KaiB does not expedite the actual phosphoryl-transfer reaction, which is never the rate-limiting step. As we were unable to stabilize the first phosphorylation site (Ser414) in the presence of ADP, the rates reported here correspond exclusively to dephosphorylation of Ser413. Second, to deconvolute the contributions of the CI and CII domains to the observed ATPase activity, we measured ADP production from KaiC_RS_ mutants that abolish hydrolysis in either the CI domain (KaiC_RS_-E62Q/E63Q) or the CII domain (KaiC_RS_-E302Q/E303Q). For wild-type KaiC_RS_, the binding of KaiB_RS_ resulted in a 7.5-fold increase in ATPase activity, and both domains were affected and contributed additively (3-fold for CI and at least 1.7-fold for CII) to the overall effect (Fig. 3d and Extended Data Fig. 8b–d). Of note, the fold increase in the CII domain represents a lower limit as the mutations induced to generate KaiC_RS_-E62Q/E63Q interfere with KaiB_RS_ binding, as previously reported for KaiC_SE_ (ref. ^30^). Third, our measurements of nucleotide exchange showed that this rate is also unaffected by KaiB_RS_ binding (19.8 ± 1.8 h^−1^ and 18.0 ± 1.5 h^−1^ with or without KaiB_RS_, respectively; Fig. 3g). As there is no tryptophan residue near the nucleotide-binding site in the CI domain, only the exchange rate in the CII domain could be determined. Notably, the change in fluorescence amplitude was smaller in the presence of KaiB_RS_, which demonstrates that even though the binding of KaiB_RS_ does not accelerate nucleotide exchange, it appears to induce a conformational rearrangement in the CII domain, especially at higher temperatures (Fig. 3g and Extended Data Fig. 8f–h).

## Structure of the KaiBC_RS_ complex

**Fig. 4:**
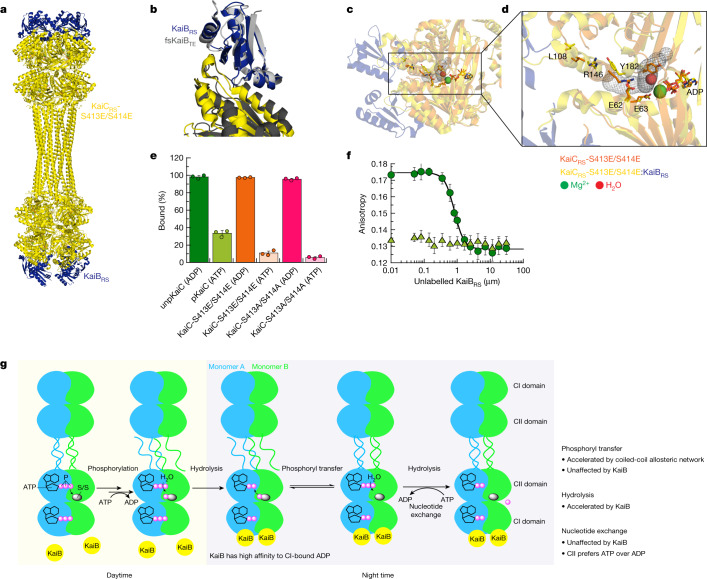
KaiB_RS_ binds to the post-hydrolysis state and accelerates the ATPase activity of KaiC_RS_. **a**, Cryo-EM structure of KaiC_RS_-S413E/S414E (yellow) in complex with KaiB_RS_ (blue) (PDB: 8FWJ). **b**, Superposition of KaiC_RS_-S413E/S414E (yellow) bound to KaiB_RS_ (blue) (PDB: 8FWJ) and KaiC_TE_-S413E (dark grey) bound to fsKaiB_TE_ (fold-switch, binding-competent state of KaiB_TE_; light grey) (PDB: 5JWQ)^26^. **c**,**d**, Binding of KaiB_RS_ (blue) creates a tunnel (grey mesh) that enables water to reach the catalytic position (red sphere) for ATP hydrolysis in the CI domain. **e**, Binding of wild-type and mutant forms of KaiC_RS_ to His-tagged KaiB_RS_ in the presence of ADP or an ATP-recycling system at 25 °C. Bar graphs show mean ± s.d. from three replicates. **f**, Fluorescence anisotropy of unlabelled KaiB_RS_ competitively displacing KaiB_RS_–6IAF (where 6IAF is the fluorophore) from unphosphorylated KaiC_RS_ in the presence of ADP (dark green circles) and phosphorylated KaiC_RS_ in the presence of the ATP-recycling system (light green triangles) at 30 °C. The average anisotropy and standard error were calculated from ten replicate measurements. **g**, Schematic of the uncovered mechanism of KaiC_RS_ regulated by coiled-coil interactions and KaiB_RS_ in the CI and CII domains.

**Extended Data Fig. 9:**
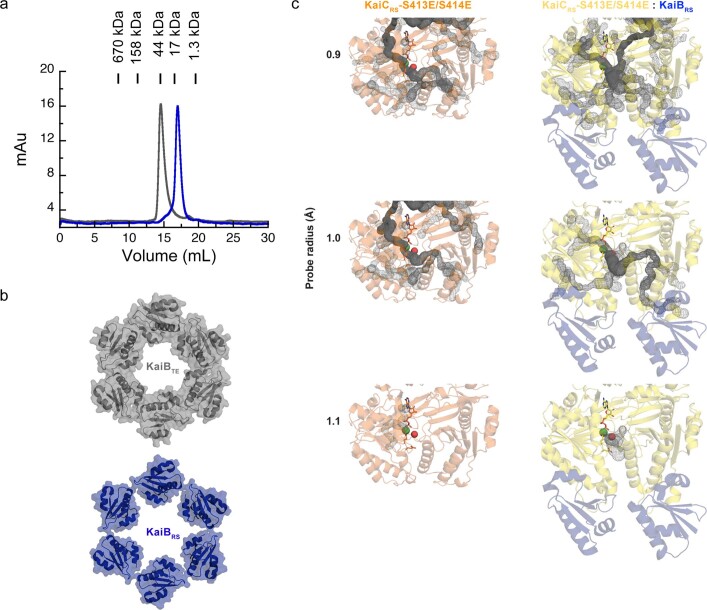
KaiB-KaiB interface in the KaiBC_RS_ complex affects the solvent accessibility into the active site of KaiC_RS_-CI. (**a**) Size-exclusion chromatography of KaiB_RS_ (blue) shows that it is monomeric in solution in contrast to KaiB_SE_ (gray), which elutes as a tetramer. Molecular-weight standards are shown above the chromatogram. **(b)** Structural comparison of KaiB_TE_ (gray, PDB 5jwq^26^) and KaiB_RS_ (blue) when bound to their corresponding KaiC hexamers. The PISA software package^37^ determines that for the KaiBC_TE_ complex the interface between the KaiB_TE_ monomers is 255 Å^2^, whereas the average interface between KaiB_RS_ monomers is only 45 Å^2^ in the KaiBC_RS_ complex. **(c)** To understand how KaiB_RS_ binding to KaiC_RS_-CI domain increases the hydrolysis rate, we investigated whether conformational changes modulated substrate access to the active site. The CAVER software^61^ was used to calculate tunnels (gray mesh) leading into the active site of the CI domain of KaiC_RS_-S413E/S414E alone (orange) and the KaiC_RS_-S413E/S414E:KaiB_RS_ complex (yellow:blue) with varying probe radii. In both structures, the active site was occupied by ADP:Mg^2+^ (sticks and green sphere, respectively). The crystal structure of bovine F1-ATPase in complex with a transition-state analogue (PDB 1w0j, chain D)^62^ was used as a reference to determine the position of the catalytic water molecule in the active site (shown as a red sphere). The calculated tunnels connect bulk solvent to the catalytic water when KaiB_RS_ is bound for probe radii larger than the default value of 0.9 Å, but never in its absence. These results suggest that KaiB_RS_ facilitates the access of water into the active site of KaiC_RS_-CI via long-range conformational changes and thus enhances ATP hydrolysis.

**Extended Data Fig. 10:**
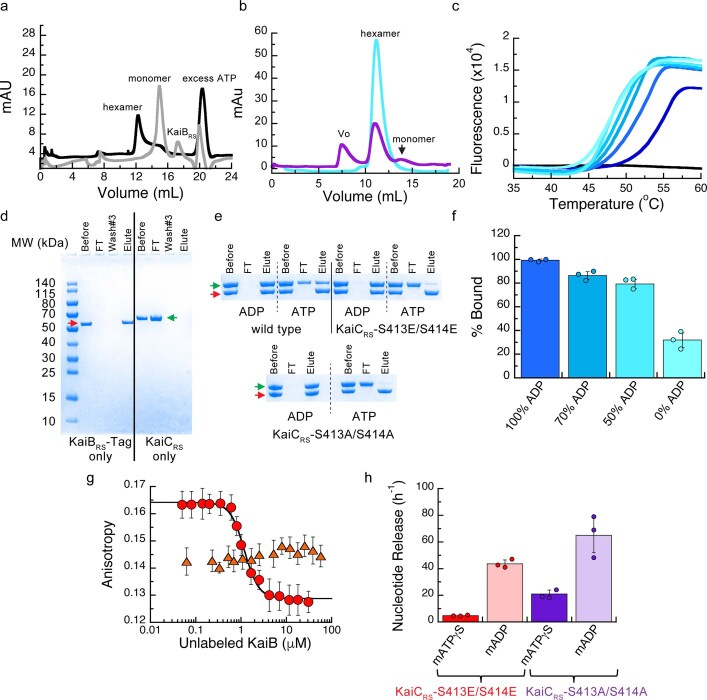
KaiB_RS_ binds preferentially to the post-hydrolysis state of KaiC_RS_ and affects its stability. **(a)** Size-exclusion chromatography of 50 μM KaiC_RS_-CI (CI_RS_ domain) in the absence (black line, hexamer) and presence (gray line, monomer) of 50 μM KaiB_RS_ in 1 mM ATP buffer. **(b)** Size-exclusion chromatography of 50 μM KaiC_RS_-Δcoil in the presence of 50 μM KaiB_RS_ (purple). The reference sample (50 μM KaiC_RS_-Δcoil) is a hexamer in solution (cyan) and after the addition of 50 μM KaiB_RS_ the mixture was incubated at 30 °C for 3.5 h (purple) before running the samples again on a Superdex-200 10/300 GL column at 4 °C. These data show that binding of KaiB_RS_ results in (i) disassembly of the hexameric KaiC_RS_-Δcoil structure into its monomers and (ii) aggregation as detected by the elution in the void volume of the column (v_0_). **(c)** Thermal denaturation profiles for KaiC_RS_-S413E/S414E in the presence of 1 mM ADP are shown from dark to light blue for increasing concentrations of KaiB_RS_ (between 0 – 4 μM). The black line represents KaiB_RS_ alone (15 μM), which shows no fluorescence signal as it does not bind to SYPRO Orange due to a lack of a hydrophobic core. The T_m_ decreases upon the addition of KaiB_RS_, indicating that binding of KaiB_RS_ destabilizes the KaiC_RS_ dodecamer. Likely due to loosening up of interface and the KaiC_RS_ structure, thereby allowing for the formation of a tunnel that connects bulk solvent to the position of the hydrolytic water in the active site (see Extended Data Fig. 9). **(d)** SDS-PAGE analysis showing the control experiment for pull-down assay. The first four lanes after the molecular weight marker are KaiB_RS_-Tag samples (red arrow) and show that KaiB_RS_-Tag binds tightly to the column. The last four lanes are control pull-down assay experiments for KaiC_RS_ (green arrow) and show that KaiC_RS_ alone is unable to bind to the column. The lanes represent the initial sample used in pull-down assay (Before), flow-through after loading sample onto the column (FT), flow-through after washing the column three times with the binding buffer (Wash #3), and sample after elution with imidazole (Elute). (**e**) SDS-PAGE analysis of pull-down assay to measure the complex formation between KaiB_RS_-Tag and wild-type KaiC_RS_, KaiC_RS_-S413E/S414E, or KaiC_RS_-S413A/S414A in the presence of 4 mM ADP or ATP (with an ATP-recycling system). For gel source data, see Supplementary Figure 2. **(f)** Percentage of wild-type KaiC_RS_ bound to KaiB_RS_-Tag protein for different ATP-to-ADP ratios (4 mM total nucleotide concentration) at 25 °C as measured from pull-down assays. **(g)** Fluorescence anisotropy at 30 °C of unlabeled KaiB_RS_ competitively replacing the fluorophore-labeled KaiB_RS_ (KaiB_RS_-6IAF) from KaiC_RS_-S413E/S414E in the presence of 4 mM ADP (red circles, *K*_D_ value of 0.79 ± 0.06 μM) and 4 mM ATP with an ATP-recycling system (orange triangles). In the latter experiment no change in anisotropy is observed, indicating that only a small fraction of KaiB_RS_-6IAF is bound under these conditions. The average anisotropy and standard error were calculated from ten replicate measurements. **(h)** The mant-ATPγS or mant-ADP release is shown as bar graphs with observed rates of 4.8 ± 0.2 h^−1^ and 43.6 ± 3.0 h^−1^ for mant-ATPγS and mant-ADP releasing from KaiC_RS_-S413E/S414E, respectively, and 21.0 ± 3.0 h^−1^ and 65 ± 15 h^−1^ for mant-ATPγS and mant-ADP releasing from KaiC_RS_-S413A/S414A, respectively. The result shows that CII domain of KaiC_RS_ prefers binding of ATP over ADP. Experiments in panels (f) and (h) were performed in triplicate and data are presented as mean values ± s.d.

To elucidate the structural underpinning of the enhanced ATPase activity of KaiC_RS_ after KaiB_RS_ binding, we solved the cryo-EM structures of KaiC_RS_ alone (PDB: 8FWI) and in complex with KaiB_RS_ (PDB: 8FWJ) (Extended Data Table 2). Twelve KaiB_RS_ molecules (monomeric in solution; Extended Data Fig. 9a) bind to the CI domain of the KaiC_RS_-S413E/S414E dodecamer (Fig. 4a–c and Extended Data Fig. 9b). The bound state of KaiB_RS_ adopts the same fold-switch conformation as observed for KaiB_TE_ (ref. ^25^) and suggests that this is the canonical binding-competent state (Fig. 4b). Following binding of KaiB_RS_, the CI–CI interfaces loosen up (Fig. 4c), which enables the formation of a tunnel that connects bulk solvent to the position of the hydrolytic water in the active sites (Fig. 4d and Extended Data Fig. 9c). There are other lines of evidence for the weakened interactions within the CI domains. First, KaiB_RS_ binding to either KaiC_RS_-CI domain (Extended Data Fig. 10a) or KaiC_RS_-Δcoil (that is, missing the C-terminal extensions; Extended Data Fig. 10b) resulted in disassembly of the hexameric KaiC_RS_ structure into its monomers. By contrast, full-length KaiC_RS_ maintained its oligomeric state following binding of KaiB_RS_, which is probably due to the stabilization provided by the coiled-coil interaction. Second, a decrease in melting temperature (*T*_m_) of KaiC_RS_ was observed with increasing KaiB_RS_ concentration (Extended Data Fig. 10c). There was no interaction between neighbouring KaiB_RS_ molecules within the complex (Extended Data Fig. 9b), which suggests that there is a non-cooperative assembly of KaiB_RS_ to KaiC_RS_. This result is contrary to what has been observed for KaiBC_SE_ and KaiBC_TE_ complexes^31,32^.

Furthermore, we noted that KaiB-bound structures in phosphomimetic variants of KaiC_RS_ (Fig. 4c,d) and KaiC_SE_ (ref. ^26^) have ADP bound in their CI domain. This result demonstrates that the post-hydrolysis state is also the binding-competent state for KaiB_RS_. To test this hypothesis, a His-tagged KaiB_RS_ protein was used in pull-down assays to detect its physical interaction with wild-type and mutant forms of KaiC_RS_ bound with either ADP or ATP. Nearly all KaiB_RS_ was complexed to ADP-bound KaiC_RS_, whereas less than 30% co-eluted in the ATP-bound form, regardless of the phosphorylation state (Fig. 4e and Extended Data Fig. 10d,e). The formation of complexes depended inversely on the ATP-to-ADP ratio (Extended Data Fig. 10f). We performed fluorescence anisotropy competition experiments to obtain a more quantitative description of the binding interaction between KaiC_RS_ and KaiB_RS_. Highly similar dissociation constant (*K*_d_) values were obtained for unphosphorylated, wild-type KaiC_RS_ (Fig. 4f) and its phosphomimetic form (Extended Data Fig. 10g) bound with ADP (0.42 ± 0.03 µM and 0.79 ± 0.06 µM, respectively). No measurable binding curves were obtained for ATP-bound phosphorylated wild-type KaiC_RS_ (Fig. 4f) or for KaiC_RS_-S413E/S414E (Extended Data Fig. 10g) with ATP-recycling system, which is probably due to the small fraction of complex present. Our data show that the post-hydrolysis state in the CI domain is key for KaiB_RS_ binding, whereas the phosphorylation state of KaiC_RS_ has only a marginal effect.

In summary, we demonstrate that binding of KaiB_RS_ at the CI domain in the post-hydrolysis state facilitates the hydrolysis of transiently formed ATP after dephosphorylation of KaiC_RS_ in the CII domain (Fig. 4g). Our fluorescence experiments (Fig. 3g and Extended Data Fig. 8f) detected a conformational change in the CII domain following KaiB_RS_ binding, but we did not observe major structural changes in the cryo-EM structures. Based on the temperature dependence of the fluorescence amplitudes (Extended Data Fig. 8f), we conjecture that the inability to detect conformational differences is probably because of the low temperature. As the CII domain prefers to bind ATP over ADP (Extended Data Fig. 10h), ATP hydrolysis in the CII domain stimulated by KaiB_RS_ is particularly important to keep KaiC_RS_ in its dephosphorylated state at night time. During this period, the exogenous ATP-to-ADP ratio remains sufficiently high to otherwise result in ATP-binding in the CII active site (Fig. 3c and Extended Data Fig. 6b).

## Discussion

The KaiBC_RS_ system studied here represents a primordial, hourglass timekeeping machinery, and its mechanism provides insight into more evolved circadian oscillators such as KaiABC. The dodecameric KaiC_RS_ showed constitutive kinase activity owing to its extended C-terminal tail that forms a coiled-coil bundle with the opposing hexamer. This structure elicits a conformation akin to the exposed A loop conformation in KaiAC_SE_, and autophosphorylation occurs within half an hour. In the KaiABC_SE_ system, the transition from unphosphorylated to double phosphorylated KaiC takes place over about 12 h, and the fine-tuning of this first half of the circadian rhythm is accomplished by the emergence of KaiA_SE_ during evolution. The second clock protein, KaiB, binds the CI domain with the same fold-switched state in both systems. The interaction is controlled by the phosphorylation state in the KaiABC_SE_ system, and its sole function is to sequester KaiA_SE_ from the activating binding site, whereas KaiB binding directly accelerates ATPase activity in the KaiBC_RS_ system regardless of the phosphorylation state. The KaiBC_RS_ system requires an environmental switch in the ATP-to-ADP concentration to reset the clock. The system therefore follows the day–night schedule when nucleotide concentrations inherently fluctuate in the organism. By contrast, the self-sustained oscillator KaiABC_SE_ remains functional over a wide range of nucleotide concentrations and responds to changes in the ATP-to-ADP ratio by changing its phosphorylation period and amplitude to remain entrained with the day–night cycle^33^.

The newly reported structural fold of KaiC utilizes the versatile coiled-coil architecture as part of a long-range allosteric network that regulates KaiC_RS_ dephosphorylation. Nature uses conformational changes in coiled-coil domains for a variety of regulatory functions^34^, including the activity of the motor protein dynein in the cellular transport of cargo along the actin filament^12^. A similar register shift, although in a coiled-coil interaction formed by only two helices, is used in dynein motility. Given that this simple heptad repeat sequence emerged multiple times and is found throughout all kingdoms of life^35^, it is an example of convergent evolution.

## Methods

### Construct of KaiC and KaiB expression vectors

The wild-type KaiC_RS_ (GenBank identifier: ACM04290.1) and KaiB_RS_ (GenBank: WP_002725098.1) from *R.* *sphaeroides* strain KD131/KCTC 12085 (equivalent: *Cereibacter sphaeroides* strain KD131) constructs used in this paper were ordered from GenScript (Supplementary Table 1). Codon-optimized plasmids for KaiC_RS_ and KaiB_RS_ were subcloned into NcoI/KpnI sites of the pETM-41 vector. A QuikChange II Site-Directed Mutagenesis kit (Agilent Technologies) was used to generate single mutant, double mutant and truncated versions of KaiC_RS_. The truncated KaiC_RS_ (KaiC_RS_-Δcoil and KaiCI_RS_) were generated by introducing stop codons in the KaiC_RS_ wild-type plasmid. All primers were ordered from Genewiz (Supplementary Table 2). The presence of the intended KaiC_RS_ mutations in the plasmid was confirmed by DNA sequencing by Genewiz using primers ordered from the same company (listed in Supplementary Table 2).

Both KaiC_SE_ and KaiA_SE_ plasmids were a gift from E. K. O’Shea. Expression and purification were performed according to a previously described procedure^6^.

### Expression and purification of KaiC_RS_ and KaiB_RS_ from *R.sphaeroides*

KaiC_RS_, KaiC_RS_ mutants and KaiB_RS_ were expressed in *Escherichia coli* BL21(DE3) cells (New England Biolabs) harbouring the plasmid pETM-41 containing the *kaiC*_*RS*_ or *kaiB*_*RS*_ gene. Three colonies from a freshly prepared transformation plate were inoculated into 1 litre of TB medium containing 50 μg ml^–1^ kanamycin. This culture was grown at 25 °C with shaking at 220 r.p.m. for 48 h without IPTG induction (leaky expression). The cells were pelleted by centrifugation at 4,200 r.p.m. for 15 min at 4 °C and stored at −80 °C.

Frozen cell pellets of KaiC_RS_ and KaiC_RS_ mutants were resuspended into lysate buffer (buffer A_C-RS_) containing 1× EDTA-free protease inhibitor cocktail (Thermo Fisher Scientific), DNAse I (Sigma Aldrich) and lysozyme (Sigma Aldrich), and the lysate was sonicated for 10–15 min (20 s on, 30 s off, output power less than 40%) on ice followed by centrifugation at 18,000 r.p.m. at 4 °C for 45 min to remove cell debris. The lysate was filtered through a 0.45 μm filter and then loaded on HisTrap HP prepacked Ni-sepharose columns (Cytiva) pre-equilibrated with buffer A_C-RS_ at 0.5 ml min^–1^. The column was washed with buffer A_C-RS_ at 1 ml min^–1^ until the UV absorbance returned to baseline. Impurities were then washed with 15% buffer B_C-RS_, and the protein was eluted with 50% buffer B_C-RS_. The eluted complex was diluted with 1.5-fold dialysis buffer_C-RS_ then subjected to in-house prepared His-tagged TEV protease (1:10, TEVP:KaiC_RS_ molar ratio) cleavage to remove the His_6_–MBP tag from KaiC_RS_ (wild-type and mutants) overnight at 4 °C in 6–8 kDa snakeskin dialysis tubing (Thermo Fisher Scientific) that was exchanged against dialysis buffer_C-RS_. Cleaved KaiC_RS_ was filtered through a 1 μm filter and once again loaded onto HisTrap HP prepacked Ni-sepharose columns at 0.5 ml min^–1^ to remove His-tagged TEV protease, His_6_–MBP tag and uncleaved protein. The flow through was concentrated using a Millipore Amicon Ultra-15 centrifugal filter device (10 kDa cut-off) and immediately passed through a HiPrep Sephacryl S-400 HR column (Cytiva) pre-equilibrated with buffer C_C-RS_. Protein was purified to homogeneity with a single band on Bis-Tris 4–12% gradient SDS–PAGE gel (Genscript) at 62.5 kDa. All protein purification steps were done at 4 °C or on ice. Protein was aliquoted and flash-frozen before storage at −80 °C until further use. The protein concentration was measured using a Microplate BCA Protein Assay kit (Thermo Fisher Scientific) on a SpectraMax MiniMax 300 imaging cytometer using BSA as a standard curve. Typical yields of KaiC_RS_ (wild-type and mutants) were 20–40 mg l^–1^ of culture.

To test whether the purified KaiC_RS_ had any ATPase contamination, Q-sepharose HP columns (Cytiva) pre-equilibrated with buffer D_C-RS_ was used before the final HiPrep Sephacryl S-400 HR column. The protein was eluted with 5 CV of a linear gradient from 0 to 100% buffer E_C-RS_. The ATPase activity of the protein samples purified using Q-sepharose HP columns was identical to the samples without this additional purification step. Buffer A_C-RS_ comprised 50 mM Tris-base (pH 7.5), 250 mM NaCl, 10 mM imidazole, 2 mM TCEP, 5 mM MgCl_2_, 1 mM ATP and 10% glycerol (v/v). Buffer B_C-RS_ comprised 50 mM Tris-base (pH 7.5), 250 mM NaCl, 500 mM imidazole, 2 mM TCEP, 5 mM MgCl_2_, 1 mM ATP and 10% glycerol (v/v). Dialysis_C-RS_ comprised 50 mM Tris-base (pH 7.0), 50 mM NaCl, 2 mM TCEP, 5 mM MgCl_2_, 1 mM ATP and 10% glycerol (v/v). Buffer C_C-RS_ comprised 50 mM Tris-base (pH 7.0), 50 mM NaCl, 2 mM TCEP, 5 mM MgCl_2_, 1 mM ATP and 10% glycerol (v/v). Buffer D_C-RS_ comprised50 mM Tris-base (pH 7.0), 2 mM TCEP, 5 mM MgCl_2_, 1 mM ATP and 10% glycerol (v/v). Buffer E_C-RS_ comprised 50 mM Tris-base (pH 7.0), 1 M NaCl, 2 mM TCEP, 5 mM MgCl_2_, 1 mM ATP and 10% glycerol (v/v).

The purification of KaiB_RS_ was similar to KaiC_RS_, but with slight modifications as outlined below. After sonication and centrifugation to remove cell debris, the lysate was filtered through a 0.22 μm filter and then passed through HisTrap HP prepacked Ni Sepharose columns, pre-equilibrated with buffer A_B-RS_. The column was washed with buffer A_B-RS_ until the UV absorbance returned to baseline. Impurities were washed with 5% buffer B_B-RS_, and the protein was eluted with 50% buffer B_B-RS_. The fusion protein was concentrated down to around 30 ml using Amicon stirred cells (Millipore Sigma) with 10 kDa cut-off. In-house prepared His-tagged TEV protease was added, and the fusion protein was cleaved overnight at 4 °C in a 3.5 kDa dialysis cassette that was exchanged against dialysis_B-RS_ buffer. The cleaved KaiB_RS_ was passed through HisTrap HP prepacked Ni-sepharose columns and concentrated down to about 10 ml using a Millipore Amicon Ultra-15 centrifugal filter device (3.5 kDa cut-off). The protein sample was then loaded onto a 26/60 Superdex S75 gel-filtration column (Cytiva) pre-equilibrated with buffer C_B-RS_ at 4 °C. The eluted protein was loaded onto Q-sepharose HP columns pre-equilibrated with buffer D_B-RS_ to remove ATPase contamination. The protein was eluted out in the flow through. A gradient from 0 to 100% buffer E_B-RS_ was passed through Q-sepharose HP columns to ensure that no KaiB_RS_ was bound to the columns. Protein was purified to homogeneity with the single band on Bis-Tris 4–12% gradient SDS–PAGE gels at 10.3 kDa. Protein was aliquoted and flash-frozen before storage at −80 °C until use. The protein concentration was measured using a Microplate BCA Protein Assay kit (Thermo Fisher Scientific) on a SpectraMax MiniMax 300 imaging cytometer using BSA as a standard curve. Typical yields of KaiB_RS_ were 30–40 mg l^–1^ of culture.

Buffer A_B-RS_ comprised 50 mM Tris-base (pH 7.5), 250 mM NaCl, 10 mM imidazole, 2 mM TCEP and 10% glycerol (v/v). Buffer B_B-RS_ comprised 50 mM Tris-base (pH 7.5), 250 mM NaCl, 500 mM imidazole, 2 mM TCEP and 10% glycerol (v/v). Dialysis_B-RS_ comprised 50 mM Tris-base (pH 7.5), 250 mM NaCl, 2 mM TCEP and 10% glycerol (v/v). Buffer C_B-RS_ comprised 50 mM Tris-base (pH 7.5), 50 mM NaCl, 2 mM TCEP and 10% glycerol (v/v). Buffer D_B-RS_ comprised 50 mM Tris-base (pH 7.5), 2 mM TCEP and 10% glycerol (v/v). Buffer E_B-RS_ comprised 50 mM Tris-base (pH 7.5), 1 M NaCl, 2 mM TCEP and 10% glycerol (v/v).

### Phylogenetic tree of KaiC

Protein sequences used in this study were identified in a multistep process. In the first step, a selection of sequences was identified using the BLASTP algorithm, utilizing a query based on the protein sequence for KaiC from *S.* *elongatus* (GenBank: WP_011242648.1)^38^. The query was run against NCBI’s non-redundant protein database with the exclusion of models or uncultured and environmental sample sequences. A multiple sequence alignment of the selected 1,538 sequences was generated using MAFFT^39–41^ (Supplementary Dataset 1). This alignment was used as input to generate an initial phylogenetic tree for KaiC with RAxML (v.8.2.9)^42^ using the PROTGAMMALG model. The generated tree was then used to identify the emergence of KaiB to create the final tree that focused on systems containing either KaiBC or KaiABC. To do so, a BLASTP search was performed for each branch tip with the KaiB sequence from *S.* *elongatus*, with the results restricted to the organism at which the branch tip was identified from. The observed branch point of emergence of KaiB agrees well with previous results in which it was shown that KaiB is mainly seen in non-archaea, non-proteobacteria^2^.

To ensure the best possible sequence coverage, a BLASTP search using KaiB as query (GenBank: WP_011242647.1) was performed. The resulting sequences were then used to identify the organisms that they came from, which allowed us to create a list of organisms with an identified KaiB sequence. This list was then used to select for a subsequent BLASTP search using KaiC as query and therefore to identify only KaiC sequences for organisms that contain both KaiB and KaiC. A spot check was run to confirm that, for example, KaiA was indeed found among all cyanobacteria identified except for *Prochlorococcus marinus*. The obtained sequences were trimmed down to only include sequences with a sequence homology of 90% or less using CD-HIT^43^ to arrive at a total of 401 sequences. For the calculation of the phylogenetic tree, RecA from *S.* *elongates* was added to serve as the outgroup. Sequences were aligned using MAFFT^39–41^ (Supplementary Dataset 2) with the E-INS-I algorithm^44^. The multiple sequence alignment was then used as input for the phylogenetic tree calculation with IQ-TREE (v.1.6.beta5), using the LG-substitution matrix^45^ with the freeRate model (using 10 categories; LG+R10)^46,47^. To enable determination of branch support, an aBayes test^48^, a SH-aLRT test (20,000 bootstrap replicates^49^) and an ultrafast bootstrap (20,000 bootstrap replicates^50^) were performed (Supplementary Dataset 1; branch supports in order: SH-aLRT support (%)/aBayes support/ultrafast bootstrap support (%)).

### X-ray crystallography

KaiC_RS_ and KaiC_RS_-Δcoil crystals were obtained by sitting-drop vapour diffusion using a 96-well Intelli-Plate (102-0001-00, Art Robbins) at 291 K. Drops contained 0.5 μl crystallization solution, 0.5 μl protein at 10 mg ml^–1^ in 20 mM MOPS pH 6.5, 50 mM NaCl, 2 mM TCEP, 5 mM MgCl_2_, 3 mM ATP and 1 mM AMPPCP, and were equilibrated against 50 μl of the solution in the reservoir. The KaiC_RS_ crystallization solution consisted of 200 mM magnesium chloride hexahydrate, 100 mM HEPES pH 7.5 and 30% (w/v) PEG 400. KaiC_RS_-Δcoil crystals were grown using 200 mM ammonium acetate, 100 mM sodium citrate tribasic dihydrate pH 5.6 and 30% (w/v) PEG 4,000.

The PEG 400 in the KaiC_RS_ crystallization solution acted as a cryoprotectant, whereas KaiC_RS_-Δcoil crystals were cryoprotected in LV Cryo Oil (MiTeGen). Single crystals were cooled in liquid nitrogen, and X-ray diffraction images were collected at ALS beamline 8.2.1 at 100 K (data collection details are described in Extended Data Table 1). The data were indexed and integrated in iMosflm^51^, and scaled and merged in Aimless^52^.

To obtain a structural model of KaiC_RS_, first the KaiC_RS_-Δcoil structure was solved by molecular replacement in MRage^53^ using the KaiC_RS_ sequence (residues 1–490) as input to search for homologues in the PDB database. The initial KaiC_RS_-Δcoil structure based on the KaiC_SE_ (PDB: 1TF7 (ref. ^36^)) was manually rebuilt in Coot (v.0.9.81)^54^ and refined in Phenix (v.1.20.1-4487)^55^. Finally, the KaiC_RS_-Δcoil structure was used as the molecular replacement search model in Phaser^56^ to solve the full-length KaiC_RS_ structure.

The assigned space groups were validated in Zanuda^57^, and the position of the asymmetric unit in the unit cell was standardized using Achesym^58^. KaiC_RS_ coiled-coil registers were analysed using SamCC-Turbo (v.0.0.2) with the default socket cut-off value of 7.4 (ref. ^59^). The images of protein structures were rendered using PyMOL (v.2.6.0)^60^.

Tunnel detection and calculation were performed using CAVER 3.0.2 PyMOL plugin^61^, with the minimum probe radius varying between 0.9 and 1.1. Default values were used for all other parameters. All atoms except waters were used in the calculation. The residue selection for starting point consisted of Glu62, Glu63 and ADP602. The catalytic position of the water in the CI domain was modelled from the crystal structure of the transition-state analogue-bound F_1_-ATPase (PDB: 1w0j, water 2,064 from chain D^62^).

### Cryo-EM and image processing

For preparation of EM grids, 3–4 μl of 4.3 mg ml^–1^ (per monomer concentration) of sample in 20 mM MOPS pH 6.50, 50 mM NaCl, 2 mM TCEP, 10 mM MgCl_2_ and 2 mM ATP was applied to glow-discharged 1.2/1.3 400 mesh C-flat carbon-coated copper grids (Protochips). The grids were frozen using a Vitrobot Mark IV (ThermoFisher) at 4 °C and 95% humidity, with a blot time of 4 s. All datasets were collected on a Titan Krios operated at an acceleration voltage of 300 keV, with a GIF quantum energy filter (Gatan) and a GATAN K2 Summit direct electron detector controlled by SerialEM^63^.

Inspection of the raw cryo-EM images revealed some heterogeneity in the relative orientations between individual hexamers of the dodecameric particles, presumably due to inherent flexibility in the coiled-coil regions, which limited the resolution to 3.3–3.4 Å. To obtain higher resolution reconstructions, the dodecamers were split and processed as individual hexamers, with *C*6 symmetry being applied throughout processing. To reconstitute the full dodecamer reconstruction, two copies of the hexamer reconstruction were overlaid on top of each other using the ‘fit in map’ function in Chimera^64^ to fit one hexamer into the lower resolution end density of the other. The overlaid hexamers were then combined, creating a new map in which each voxel takes the value from the hexamer with highest absolute value.

For KaiC_RS_-S413E/S414E alone, a dataset of approximately 2,500 movies was collected. The movies were recorded with a pixel size of 1.074 Å, including 70 frames and with an exposure rate of 1.31 e^−^ per Å^2^ per frame. Approximately 825,000 particles were picked, and after 2D classification, around 320,000 particles from good class averages were carried forward for further processing. The final measured resolution of the reconstruction was 2.9 Å (Extended Data Fig. 4a).

For the KaiC_RS_-S413E/S414E:KaiB_RS_ complex, a dataset of around 2,000 movies was collected. The movies were recorded with a pixel size of 1.023 Å, including 70 frames and with an exposure rate of 1.35 e^−^ per Å^2^ per frame. About 440,000 particles were picked, and after 2D classification around 190,000 particles from good class averages were carried forward for further processing. The final measured resolution of the reconstruction was 2.7 Å (Extended Data Fig. 4b).

All data processing was carried out using *cis*TEM (v.2.0.0)^65^, and followed the workflow of motion correction, CTF parameter estimation, particle picking, 2D classification, ab initio 3D map generation, 3D refinement, 3D classification, per-particle CTF refinement and B-factor sharpening. The highest resolution of 3D refinement used was 4 Å for both reconstructions, and final resolutions were estimated using the *cis*TEM PartFSC and a threshold of 0.143.

To validate the combined dodecamer structure, we also processed both datasets as full *D*6 symmetric dodecamers (Extended Data Fig. 4c,d). This was accomplished by extracting the picked hexamers into a large box size and performing 2D classification with automatic centring. Clear dodecamer class averages were then selected and re-extracted from the original images, with picking coordinates that were adjusted by the translation required to match the centred class average. After this centring, duplicate picks were removed to obtain the final dodecamer particle stacks. These stacks were processed as described above, with the highest resolution of 3D refinement used as 4.25 Å.

In an attempt to find deviations from *D*6 symmetry, we also calculated reconstructions for both structures assuming *C*1 symmetry, starting from the ab initio 3D step. The resulting refined *C*1 structures did not exhibit detectable departures from D6 symmetry (Extended Data Fig. 4e). We therefore present symmetrized volumes as our final result.

The cryo-EM structures were built using the KaiC_RS_ model obtained by X-ray crystallography and fold-switch-stabilized KaiB_TE_ (PDB: 5JWO (ref. ^26^)) as starting points. The models were constructed using Coot (v.0.9.81)^54^, and refinement was carried out using Phenix (v.1.20.1-4487)^55^.

### Preparation of unphosphorylated KaiC_RS_

Purified KaiC_RS_ (about 20 μM) from −80 °C was dialysed in 20 mM MOPS (pH 6.5), 50 mM NaCl, 2 mM TCEP, 10 mM MgCl_2_ and 0.1 mM ADP overnight at 4 °C to remove glycerol and to replace ATP with ADP. The dialysed KaiC_RS_ was then heated at 30 °C for 4 h to obtain fully unphosphorylated KaiC_RS_ bound with ADP, and the sample was then passed through 0.22 μm Spin-X centrifuge tube filters (Corning). The sample was concentrated to a higher concentration (less than 100 μM) at 4 °C. The protein concentration was measured using a BCA assay.

### In vitro KaiBC_RS_ reaction

#### Kinetics of KaiC_RS_ autophosphorylation in the presence and absence of KaiB_RS_

Unphosphorylated KaiC_RS_ (3.5 μM, prepared as described above) in the presence or absence of KaiB_RS_ (3.5 μM and 35 μM) was preincubated at 20, 25, 30 and 35 °C for 1 h in 20 mM MOPS (pH 6.5), 50 mM NaCl, 2 mM TCEP, 10 mM MgCl_2_ and 0.1 mM ADP. The reactions were started by adding 3.9 mM ATP to obtain a final concentration of 4 mM nucleotide in the presence of 2 U ml^–1^ pyruvate kinase (Millipore Sigma) and 10 mM phosphoenolpyruvate (Millipore Sigma) to regenerate ATP during the reaction. The reaction samples were sampled by hand at specific time points and mixed with an equal amount of loading dye (stock concentration of 0.1 M Tris-base (pH 7.5), 4% SDS, 0.2% bromophenol blue, 30% glycerol and 0.5 M 2-mercaptoethanol). The mixed samples were then stored at −20 °C until further use.

#### Kinetics of KaiC_RS_ autodephosphorylation in the presence and absence of KaiB_RS_

Purified KaiC_RS_ (around 20 μM) was dialysed in reaction buffer containing 20 mM MOPS (pH 6.5), 50 mM NaCl, 2 mM TCEP, 10 mM MgCl_2_ and 0.1 mM ADP overnight at 4 °C to remove glycerol and to generate KaiC_RS_ bound with ADP. After dialysis at 4 °C, KaiC_RS_ exists in two states: 50% unphosphorylated and 50% single phosphorylated at Ser413 (pSer413), which were confirmed by tandem mass spectrometry (data not shown). The autodephosphorylation reaction was started by adding KaiC_RS_ or KaiC_RS_ in the presence of KaiB_RS_ (3.5 μM) into reaction buffer pre-equilibrated at 30 °C.

#### Oscillation of KaiBC_RS_

Dialysed KaiC_RS_ (3.5 μM) was preincubated at 35 °C for 30 min in 20 mM MOPS (pH 6.5), 50 mM NaCl, 2 mM TCEP, 10 mM MgCl_2_ and 0.1 mM ADP in the presence or absence of KaiB_RS_ (3.5 μM). The reactions were started by adding 4 mM ATP and reaction samples were collected at specific time points for 10% SDS–PAGE and HPLC analysis to identify phosphorylation state of KaiC_RS_ and amount of nucleotide at each time point, respectively.

#### Controlling ATP-to-ADP ratio to mimic daytime and night time

KaiC_RS_ was dialysed in reaction buffer containing 20 mM MOPS (pH 6.5), 50 mM NaCl, 2 mM TCEP, 10 mM MgCl_2_ and 1 mM ATP overnight at 4 °C. To start the reaction as shown in Fig. 3c, KaiC_RS_ (3.5 μM) in the absence or presence of KaiB_RS_ (3.5 μM) was mixed with additional ATP (final 4 mM to mimic daytime), and the reaction samples (500 μl) were added into a D-Tube Dialyzer (midi 3.5 kDa cut-off, EMD Millipore) that was exchanged against 4 mM ATP buffer (400 ml). After the 12-h time point, the reaction samples were transferred into preincubated 25% ATP/ADP buffer (400 ml) that mimics the night time. After the 24-h time point, the same samples were changed into preincubated 4 mM ATP to mimic the daytime again.

To start the experiment as shown in Extended Data Fig. 6a, KaiC_RS_ (35 μM) in the presence of 3 mM ATP in 20 mM MOPS (pH 6.5), 50 mM NaCl, 2 mM TCEP and 10 mM MgCl_2_ was heated at 35 °C for 25 min to generate fully phosphorylated KaiC_RS_. The KaiC_RS_ sample was then diluted 10-fold into 25% ATP/ADP buffer pre-equilibrated at 30 °C to final concentration of KaiC_RS_ (3.5 μM) and KaiB_RS_ (3.5 μM or 35 μM). The reaction samples (300 μl) were added into a D-Tube Dialyzer (midi 3.5 kDa cut-off, EMD Millipore) that was exchanged against 25% ATP/ADP buffer (300 ml).

During the reaction, the samples were gently shaken in a 30 °C incubator, and reaction samples were collected at specific time points for 10% SDS–PAGE and HPLC analysis to identify the phosphorylation state of KaiC_RS_ and the amount of nucleotide at each time point, respectively.

The rationale for the ATP-to-ADP ratio at daytime and night time comes from two earlier literature reports. The change in ATP-to-ADP ratio at daytime and night time were directly measured in vivo in the strain *R.* *sphaeroides*^27^, in which ATP is 2.0–2.4 mM during day and drops to 0.5–0.6 during night, and it is well known that the total nucleotide concentration stays constant. We chose the total nucleotide concentration of 4 mM in our in vitro work to be identical to the described in vitro experiments performed for the canonical KaiC_SE_. Because of photosynthesis in daylight, virtually all nucleotide is ATP^33^. We note that a slightly higher amount of ATP will not affect our results, as the affinity of KaiC_RS_ for ATP is higher than for ADP.

### Separation of unphosphorylated, single and double phosphorylated KaiC_RS_ by SDS–PAGE

Unphosphorylated, single phosphorylated and double phosphorylated KaiC_RS_ were separated by 10% SDS–PAGE with 37.5:1 acrylamide:bis-acrylamide (Bio-Rad), 18 cm × 16 cm × 1 mm Tris-HCl gel with 1× Tris-glycine SDS running buffer (Invitrogen). The samples were heated at 95 °C for 3 min, and 400 ng of material was loaded onto the Tris-HCl gel. The gel was run with a constant current of 35 mA, 150 W, and the voltage was greater than 700 V for 5.5 h in a cold room, with a water bath set to 12 °C using a Hoefer SE600 electrophoresis unit.

Unphosphorylated and phosphorylated KaiC_RS_-Δcoil were separated by Zn^2+^ Phos-tag SDS–PAGE with 10% acrylamide gel containing 50 μM Phos-tag acrylamide (Wako). The gel was run with a constant current of 30 mA for 5 h 30 min in a cold-room, with 1 μg per well protein samples pre-heated at 95 °C for 3 min.

The gels were stained overnight at room temperature with InstantBlue protein gel stain (Expedeon) with gentle shaking and destained with distilled water until bands were clearly visible. The gels were imaged on a ChemiDoc Imager (Bio-Rad), and Image Lab software (Bio-Rad) was used for analysis.

### Statistics and reproducibility for gel electrophoresis

Data shown in main text figures and Extended Data figures are representative SDS–PAGE gels for at least three independent biological replicates (*n* = 3), except for experiments presented in Fig. 3a,c, which were performed in duplicate.

### Oligomerization state of KaiC_RS_ and KaiB_RS_

#### Gel-filtration chromatography

KaiC_RS_, unphosphorylated KaiC_RS_ and all KaiC_RS_ mutants with a concentration of around 40–80 μM were loaded with a flow rate of 0.2 ml min^–1^ onto a prepacked Superdex-200 10/300 GL (GE Healthcare) pre-equilibrated with 20 mM MOPS (pH 6.5), 50 mM NaCl, 2 mM TCEP, 10 mM MgCl_2_ and 1 mM ATP (0.1 mM ADP for unphosphorylated KaiC_RS_) at 4 °C using an ÄKTA Pure system (GE Healthcare). KaiB_RS_ (0.5 mM) was loaded onto Superdex-75 10/300 GL (GE Healthcare) pre-equilibrated with 20 mM MOPS (pH 6.5), 50 mM NaCl and 2 mM TCEP at 4 °C. The eluate was collected in fractions of 1 ml each and subjected to SDS–PAGE analysis. A standard curve (that is, molecular weight versus elution time) was determined for the column using molecular weight protein standards (Bio-Rad) run in the same buffer and flow rate. The protein standard mixture contained thyroglobulin (670 kDa), γ-globulin (158 kDa), ovalbumin (44 kDa), myoglobin (17 kDa) and vitamin B_12_ (1.35 kDa).

#### Analytical ultracentrifugation

Sedimentation velocity centrifugation experiments were run at 50,000 r.p.m. (for KaiB_RS_) and 30,000 r.p.m. (for KaiC_RS_ wild-type and mutants and KaiC_SE_), with continuous scans from 5.8 to 7.3 cm at 0.005 cm intervals at 20 °C on a Beckman Optima XL-A (Beckman-Coulter) equipped with absorption optics and a four-hole An60Ti rotor. Measurements were set up at 280 nm (for KaiB_RS_) and 295 nm (for KaiC_RS_ and KaiC_SE_) to avoid interference from ATP. The software package SEDFIT (v.14.1) was used for data evaluation^66﻿^. KaiC_RS_ and KaiC_RS_-Δcoil (100 μM) were prepared in 20 mM MOPS (pH 6.5), 50 mM NaCl, 2 mM TCEP, 10 mM MgCl_2_ and 1 mM ATP. KaiC_SE_ (100 μM) was prepared in 20 mM MOPS (pH 8.0), 150 mM NaCl, 2 mM TCEP, 5 mM MgCl_2_, and 1 mM ATP. KaiB_RS_ (500 μM) was prepared in 20 mM MOPS (pH 6.5), 50 mM NaCl and 2 mM TCEP.

### ATPase activity

Purified KaiC_RS_ (both wild-type and mutant forms (around 20 μM)) and KaiB_RS_ (about 90 μM) were dialysed in 20 mM MOPS (pH 6.5), 50 mM NaCl, 2 mM TCEP, 10 mM MgCl_2_ and 1 mM ATP (reaction buffer) overnight at 4 °C. The samples were passed through 0.22 μm Spin-X centrifuge tube filters, and concentrations were measured using a BCA assay before setting up the reactions. Typical KaiC_RS_ or KaiBC_RS_ reactions contained 3.5 μM KaiC_RS_ (wild type and mutants) and 3.5 μM KaiB_RS_ in reaction buffer with a final concentration of 4 mM ATP. The samples were incubated at the indicated temperatures and were sampled by hand at specific time points. Next 10 μl of sample was quenched with 10 μl of 10% trichloroacetic acid (Millipore Sigma), and the mixture was passed through a 0.22 μm Spin-X centrifuge tube filter to remove the precipitated protein. The flow through sample was then re-adjusted to pH 6.2 for nucleotide separation by adding 10 μl of 0.75 M HEPES, pH 8.0. The final samples were kept at −20 °C until HPLC analysis.

Three microlitres of each sample were injected with a high-precision autosampler (injection error of <0.1 μl, resulting in a maximum systemic error of about 6%) to a reverse-phase HPLC instrument with an ACE 5 μm particle size, C18-AR and 100 Å pore size column (Advanced Chromatography Technologies). The instrument was pre-equilibrated with 100 mM potassium phosphate pH 6.2 with a flow rate of 0.4 ml min^–1^. Using pure nucleotide samples, the retention times of ATP, ADP and AMP were determined to be 2.6, 3.1 and 4.4 min, respectively. The concentration of each nucleotide was calculated from the relative ratio of the peak areas and the total nucleotide concentration. To determine ATPase activity rates, the observed rate constants were determined from at least five data points for each temperature using initial rate analysis and least-squares linear regression (Extended Data Figs. 6c,d and 8b–d). The mean values and uncertainties (s.d.) shown in Fig. 3d, Extended Data Figs. 6 and 8 were derived from three replicate experiments. KaleidaGraph (v.4.5.3; Synergy) was used for data analysis and plotting.

### Nucleotide exchange

KaiC_RS_ (wild type or mutants) and KaiB_RS_ were dialysed into 20 mM MOPS (pH 6.5), 50 mM NaCl, 2 mM TCEP, 10 mM MgCl_2_ and 50 μM ATP overnight at 4 °C. The samples were passed through 0.22 μm Spin-X centrifuge tube filters, and the protein concentration was measured using a BCA assay. The reaction contained 3.5 μM of KaiC_RS_-S413E and/or 35 μM of KaiB_RS_, and samples were incubated at 20, 25, 30 and 35 °C for 16–24 h in the presence of an ATP-recycling system. The reactions were started by adding 250 μM of mant-ATP (Jena Bioscience). The spectrum was measured using the fluorescence energy transfer from tryptophan residues in KaiC_RS_ to mant-ATP by exciting the sample at 290 nm (2.5 nm bandwidth) and collecting the emission intensity from 320 nm to 550 nm (5 nm bandwidth) in increments of 2 nm. To measure the nucleotide exchange rate, the maximum change in fluorescence intensity at 440 nm (Δ*F*_440 nm_) was followed for a total time of 1,800 s in 15 s increments with anti-photobleaching mode on FluoroMax-4 spectrofluorometer (Horiba Scientific) equipped with a water bath to control the temperature. There are two tryptophan residues within 5 Å from the nucleotide-binding site in the KaiCII_RS_ domain and no tryptophan residue close to the nucleotide-binding site in the KaiCI_RS_ domain, so the nucleotide exchange observed in the experiments are for the KaiCII_RS_ domain. To ensure that the exchange rate observed in the experiments are from nucleotide exchange in the KaiCII_RS_ domain, KaiCI_RS_ (which only contains the CI domain) was tested; no change in fluorescence was observed following the addition of mant-ATP (Extended Data Fig. 8h).

The experiments for KaiC_SE_ alone and with KaiC_SE_ mixed in and for KaiA_SE_ were performed in a similar way, except that KaiC_SE_ and KaiA_SE_ were dialysed in 20 mM MOPS (pH 8.0), 150 mM NaCl, 2mM TCEP, 10 mM MgCl_2_ and 50 μM ATP overnight at 4 °C. KaiA_SE_ was incubated with KaiC_SE_ for 1 h at 30 °C before adding 250 μM mant-ATP.

For the nucleotide preference experiment, KaiC_RS_-S413E/S414E and KaiC_RS_-S413A/S414A were dialysed in 20 mM MOPS (pH 6.5), 50 mM NaCl, 2 mM TCEP, 10 mM MgCl_2_ and 20 μM ADP overnight at 4 °C. KaiC_RS_-S413E/S414E or KaiC_RS_-S413A/S414A (3.5 μM) was first mixed with mant-ATPγS or mant-ADP (150 μM), and the kinetic trace at 440 nm was recorded at 30 °C. After the fluorescence trace at 440 nm reached a plateau, which indicates that the nucleotide analogue was fully bound to the protein, a 27-fold excess of ATP (4 mM) was added to displace the bound nucleotide analogue, and the decay of fluorescence intensity was recorded at 440 nm at 30 °C. The experiments were run in triplicate, and results were averaged and fitted to a single exponential decay.

Analysis was performed by fitting individual traces to an exponential equation using KinTek Explorer software^67,68﻿]^, and error bars denote the standard errors as obtained from triplicate experiments. KaleidaGraph (v.4.5.3; Synergy) was used for data plotting.

### ^32^P-ATP radioactive labelling and experiment

^32^P-labelled KaiC_RS_ was prepared by mixing unphosphorylated KaiC_RS_ (10 μM) with 0.46 μM [γ-^32^P]ATP (3,000 Ci mmol^–1^, PerkinElmer) and 500 μM ATP in 20 mM MOPS (pH 6.5), 50 mM NaCl, 2 mM TCEP and 10 mM MgCl_2_ at 35 °C for 30 min and then immediately switched to 4 °C to prevent dephosphorylation of KaiC_RS_. The ^32^P-labelled KaiC_RS_ sample was passed through Zeba spin desalting columns (Thermo Fisher Scientific) pre-equilibrated with 20 mM MOPS (pH 6.5), 50 mM NaCl, 2 mM TCEP and 10 mM MgCl_2_ twice at 4 °C. The sample was incubated with buffer containing 1 mM ADP overnight at 4 °C to obtain ^32^P-labelled KaiC_RS_ bound with ADP. The sample was passed through a final Zeba desalting column pre-equilibrated with 20 mM MOPS (pH 6.5), 50 mM NaCl, 2 mM TCEP and 10 mM MgCl_2_ at 4 °C, and the solution was then incubated with 8 mM ADP in the presence or absence of KaiB_RS_ at 4 °C for 1 h. The samples were then diluted in 20 mM MOPS (pH 6.5), 50 mM NaCl, 2 mM TCEP and 10 mM MgCl_2_ preincubated at 30 °C to obtain a final concentration of ^32^P-labelled KaiC_RS_ (5 μM), KaiB_RS_ (20 μM) and ADP (4 mM). The reactions were incubated at 30 °C, and at different time points, aliquots were taken (1.5 μl). The reactions were stopped by adding 1.5 μl Laemmli sample buffer (62.5 mM Tris (pH 6.8), 2% SDS, 25% glycerol and 0.01% bromophenol blue) supplemented with 5% (v/v) 2-mercaptoethanol.

The samples were spotted onto a TLC plate (PEI-cellulose F plates, Merck) and quickly dried with a blow-dryer for 30 s. The TLC plates were run first with distilled water as a mobile phase. After TLC plates were completely dried, 0.75 M KH_2_PO_4_ was used as the mobile phase to separate ^32^P-labelled KaiC_RS_, [γ-^32^P]ATP and inorganic phosphate (^32^P), as previously shown^29^. The phosphor-screens were scanned on an Amersham Typhoon (GE Healthcare) at a resolution of 100 μm. ImageQuant TL 7.0 software was used for analysis.

### Fluorescence anisotropy competition

Fluorescence anisotropy competition experiments were carried out using a FluoroMax-4 spectrofluorometer (Horiba Scientific) at 30 °C. Excitation and emission wavelengths for KaiB_RS_ labelled with 6-iodoacetamidofluorescein (6-IAF, Thermo Fisher Scientific) at Cys29 were set at 492 nm (5 nm bandwidth) and 520 nm (5 nm bandwidth), respectively, with fixed G factor (G factor of KaiB_RS–_6IAF alone) to eliminate instrumental bias. The average anisotropy and standard error were calculated from ten replicate measurements.

KaiB_RS_–6IAF was prepared by mixing degassed KaiB_RS_ (100 μM) with 20-fold excess of 6-IAF (stock 10 mM in 50% DMSO) in 20 mM Tris-base (pH 7.0), 50 mM NaCl and 1 mM TCEP (degassed). The reaction was incubated at room temperature for 4 h and dialysed against 20 mM Tris-base (pH 7.0), 50 mM NaCl and 1 mM TCEP in a 3.5 kDa dialysis cassette overnight at 4 °C to remove unreacted 6-IAF and small amounts of DMSO. The crosslinked sample was passed through a 0.22 μm Spin-X centrifuge tube filter and loaded onto Superdex-75 10/300 GL pre-equilibrated with 20 mM MOPS (pH 7.0), 50 mM NaCl and 1 mM TCEP with a 0.2 ml min^–1^ flow rate to remove leftover unreacted 6-IAF. The sample was aliquoted and flash-frozen in liquid nitrogen and stored at −80 °C until use. All the crosslinked reactions were performed in the dark.

For the fluorescence anisotropy competitive binding experiment, KaiB_RS_–6IAF (0.2–0.4 μM) was first incubated with wild-type or mutant KaiC_RS_ (1 μM, 60% increase in anisotropy in comparison to KaiB_RS_–6IAF alone) in 20 mM MOPS (pH 6.5), 50 mM NaCl, 2 mM TCEP and 10 mM MgCl_2_ in the presence of 4 mM ADP or 4 mM ATP with an ATP-recycling system (2 U ml^–1^ pyruvate kinase and 10 mM phosphoenolpyruvate), then unlabelled KaiB_RS_ (0–50 μM) was added to the samples. The samples were incubated at 30 °C for 4 h or 12 h before measurement.

The decrease in fluorescence anisotropy (FA) versus concentration of unlabelled KaiB_RS_ was fitted to equation (1) using the Levenberg–Marquardt nonlinear fitting algorithm included in KaleidaGraph (Synergy Software) to obtain the half-maximum inhibitory concentration (IC_50_) value. The *K*_d_ value can then be calculated from the IC_50_ value using equation (2) as previously described^69^﻿.

1 $${\rm{FA}}={m}_{1}+\frac{({m}_{2}-{m}_{1})}{1+{10}^{{\rm{\log }}x-{\rm{\log }}({{\rm{IC}}}_{50})}}$$

2 $${K}_{{\rm{d}}}=\frac{{{\rm{I}}{\rm{C}}}_{50}}{1+\frac{[{\rm{l}}{\rm{a}}{\rm{b}}{\rm{e}}{\rm{l}}{\rm{l}}{\rm{e}}{\rm{d}}\,{{\rm{K}}{\rm{a}}{\rm{i}}{\rm{B}}}_{{\rm{R}}{\rm{S}}}]}{{K}_{{\rm{d}}}}}$$

### Pull-down assays

Pull-down assays probe the interaction between KaiC_RS_ (wild type and mutants) with KaiB_RS_ (His_6_-MBP-TEV-KaiB_RS_). The His_6_-MBP-TEV-KaiB_RS_ was expressed and purified as described above but without the TEV protease cleavage step. Wild-type or mutant KaiC_RS_ (3.5 μM) was mixed with KaiB_RS_-tag (3.5 μM) in 20 mM MOPS (pH 6.5), 50 mM NaCl, 2 mM TCEP and 10 mM MgCl_2_ in the presence of 4 mM ADP or 4 mM ATP with an ATP-recycling system in a final volume of 400 μl. The samples were incubated at 25 °C for 4 h or 24 h before loading onto a 500 μl spin column with 200 μl (prepared from 400 μl of 50% slurry) Talon beads (Takara) pre-equilibrated with sample buffer. The samples were incubated with the Talon beads for 30 min with gentle shaking, after which the flow through was collected by gravity into 1 ml Eppendorf tubes. The beads were washed three times with 400 μl sample buffer by gravity, then the samples were eluted with 200 μl of 0.5 M imidazole buffer by centrifugation at 1,000*g* for 1 min. The protein mixture, flow through, wash and eluted samples were run on a Bis-Tris 4–12% gradient SDS–PAGE gel with a molecular weight marker. The gels were stained with Coomassie blue and were imaged on a ChemiDoc Imager (Bio-Rad). Image Lab Software (Bio-Rad) was used for analysis.

The following control experiments were performed: (1) fusion KaiB_RS_ protein in the absence of KaiC_RS_; and (2) KaiC_RS_ in the absence of fusion KaiB_RS_. All the fusion KaiB_RS_ proteins came out only in the elution buffer in the first control experiment, which indicated that fusion KaiB_RS_ binds to Talon beads and the amount of KaiB_RS_ used did not overload the column. All KaiC_RS_ protein came out in the flow through in the second control experiment, which indicated there is no specific binding between KaiC_RS_ and the Talon beads.

### Thermofluor assay

KaiC_RS_ and SYPRO Orange (Thermo Fisher Scientific) were used at final concentration of 3 μM and 10×, respectively. The experiments were carried out in 20 mM MOPS (pH 6.5), 50 mM NaCl, 2 mM TCEP, 10 mM MgCl_2_ and 4 mM ADP or ATP. The samples were prepared to a final volume of 20 μl in a MicroAmp Fast Optical 96-well reaction plate (Applied Biosystems Life Technologies), and the plate was sealed with Axygen UltraClear sealing film (Corning). The assay plate was run in a StepOne Real-Time PCR instrument (Applied Biosystems Life Technologies) with melt curve set up. The temperature was continuously increased from 25 °C to 95 °C by 0.3 °C every 15 s. The data were fit with nonlinear fitting in KaleidaGraph (Synergy Software) to a Boltzmann sigmoidal curve (equation (3)).

3 $$Y={\rm{Bottom}}+\frac{({\rm{Top}}-{\rm{Bottom}})}{1+{{\rm{\exp }}}^{\left(\frac{{T}_{{\rm{m}}}-T}{c}\right)}}$$

where *Y* is the fluorescence intensity at temperature *T*, *T* is the temperature in degrees Celsius, Bottom is the baseline fluorescence at low temperature, Top is the maximum fluorescence at the top of the truncated data, *c* is the slope or steepness of the curve, and *T*_m_ is the melting temperature of the protein.

### Reporting summary

Further information on research design is available in the Nature Portfolio Reporting Summary linked to this article.

## Online content

Any methods, additional references, Nature Portfolio reporting summaries, source data, extended data, supplementary information, acknowledgements, peer review information; details of author contributions and competing interests; and statements of data and code availability are available at 10.1038/s41586-023-05836-9.

### Supplementary information

Supplementary Information Supplementary Figs. 1 and 2 and Supplementary Tables 1 and 2.

Reporting Summary

Supplementary Video 1 Visualization of conformational changes in the CII domain of KaiC_RS_ after phosphorylation through the long-range allosteric network. The trajectory was made by linear interpolation between unphosphorylated KaiC_RS_ and the phosphomimetic variant (KaiC_RS_-S413E/S414E) using the morph tool in UCSF Chimera 1.15 (ref. ^64^); the movie was rendered in PyMOL (v.2.6.0)^60^.

Supplementary Data 1 Initial multiple-sequence alignment and phylogenetic tree for KaiC.

Supplementary Data 2 Final multiple-sequence alignment and phylogenetic tree including only species that contain KaiB and KaiC.

Peer Review File

## Figures and Tables

**Extended Data Table 1:**
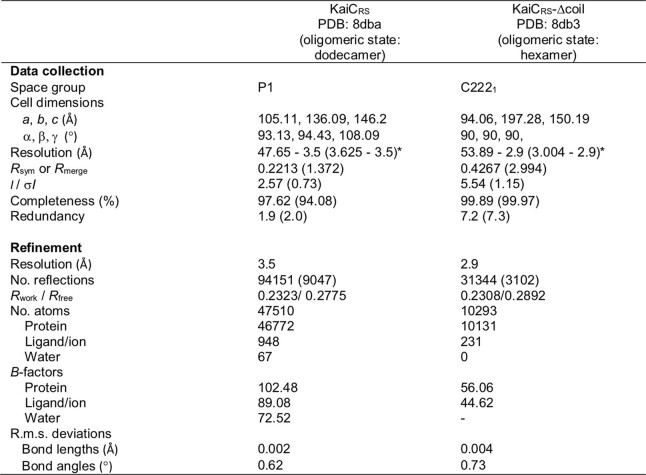
X-ray crystallography data collection and refinement statistics

A single crystal was used for each structure. *Values in parentheses are for the highest-resolution shell.

**Extended Data Table 2:**
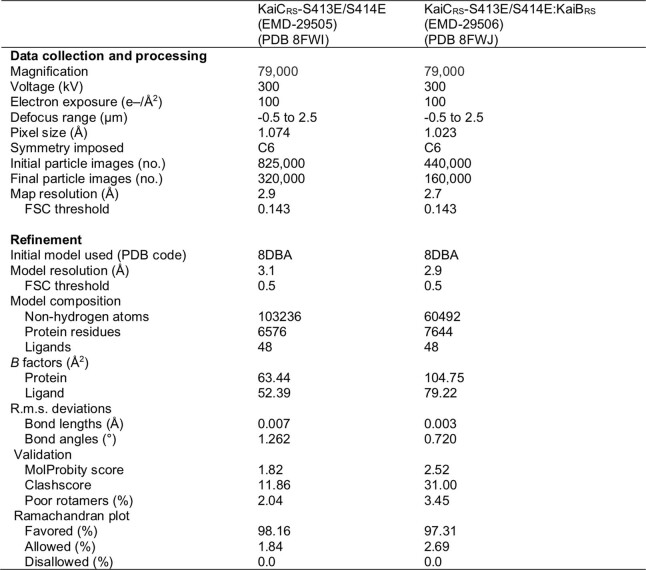
Cryo-EM data collection, refinement and validation statistics

## Data Availability

Structure factors and refined models obtained using X-ray crystallography have been deposited into PDB under accession codes 8DBA (wild-type KaiC_RS_) and 8DB3 (KaiC_RS_-∆coil). Cryo-EM maps and refined models have been deposited into the Electron Microscopy Data Bank (EMDB) and PDB, respectively. The composite map and model for the KaiC_RS_-S413E/S414E dodecamer reconstruction are submitted under entries EMD-29505 and 8FWI, respectively. The composite map and model for the KaiCRS-S413E/S414E–KaiB_RS_ dodecamer reconstruction are submitted under entries EMD-29506 and 8FWJ, respectively. The focused KaiC_RS_-S413E/S414E hexamer refinement map is available under accession EMD-29507 and the focused KaiC_RS_-S413E/S414E–KaiB_RS_ hexamer refinement map is available under accession EMD-29508. The full KaiC_RS_-S413E/S414E dodecamer refinement is available under accession EMD-29509 and the full KaiC_RS_-S413E/S414E–KaiB_RS_ dodecamer refinement is available under accession EMD-29510. Other datasets used are all publicly available in public community or discipline-specific repositories (for example, PDB identifiers 5JWQ, 1W0J, 1TF7 and 7S65). The accession codes for protein sequences, sequence alignments and phylogeny are listed in Supplementary Datasets 1 and 2.
