# Supplementary material for: From primordial clocks to circadian oscillators
Source: Nature. 2023 Mar 22;616(7955):183–9. doi: 10.1038/s41586-023-05836-9 (PMC10076222; doi:10.1038/s41586-023-05836-9)
Supplement: Supplementary file 1 — Supplementary Figs. 1 and 2 and Supplementary Tables 1 and 2. [file 41586_2023_5836_MOESM1_ESM.pdf]

nature portfolio

https://doi.org/10.1038/s41586-023-05836-9

Supplementary information

From primordial clocks to circadian oscillators

In the format provided by the authors and unedited

Supplementary Figure 1

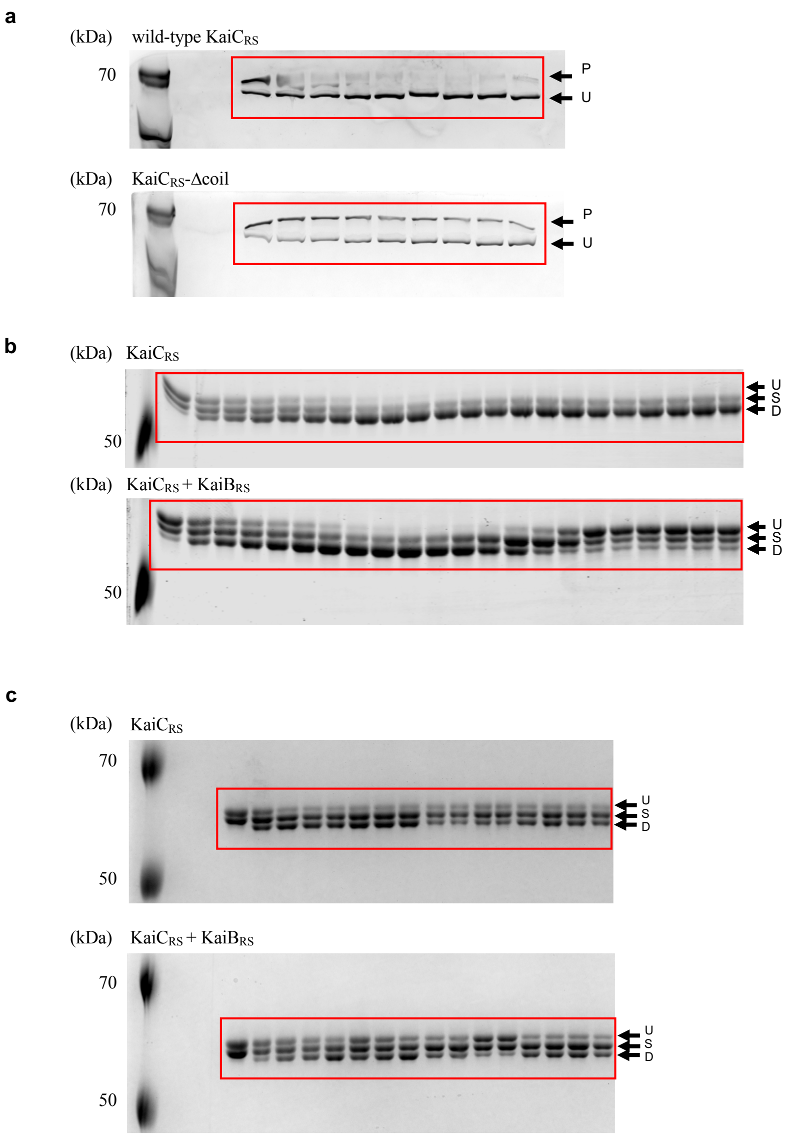

Supplementary Figure 1 | Uncropped images for SDS-PAGE gels shown in Main Text Figures. (a) Zn^{2+} Phos-tag^{TM} SDS-PAGE gel for Fig. 2f showing auto-dephosphorylation of $KaiC_{RS}$ and $KaiC_{RS}$-Δcoil over time. P and U represent phosphorylated and unphosphorylated proteins. **(b)** 10% SDS-PAGE gel for Fig. 3a showing the oscillation of $KaiC_{RS}$ in the presence of $KaiB_{RS}$ at 30 °C. **(c)** 10% SDS-PAGE gel for Fig. 3c showing the oscillation of $KaiC_{RS}$ in the presence of $KaiB_{RS}$ with the ATP-to-ADP ratio that mimic daytime and nighttime.

Supplementary Figure 2

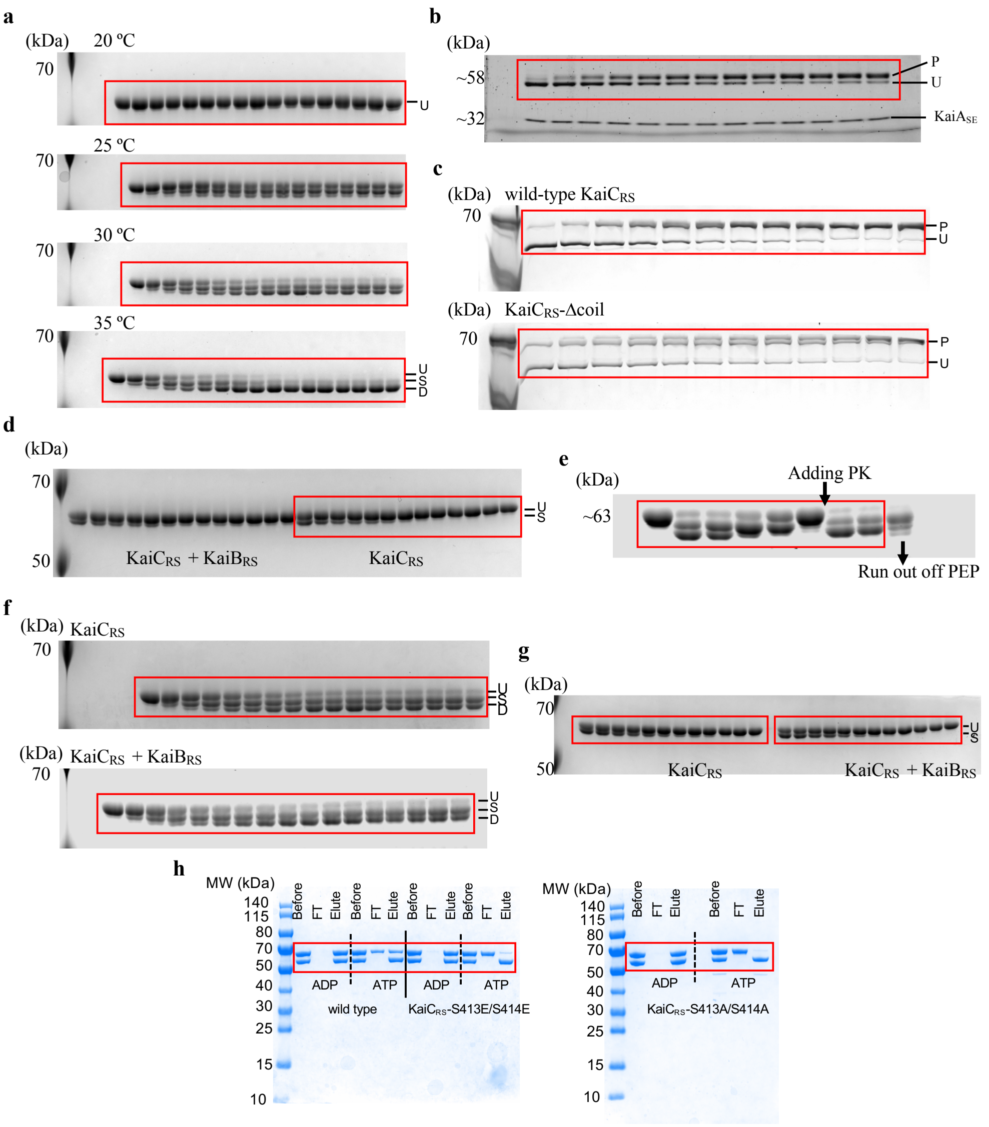

Supplementary Figure 2 | Uncropped images for SDS-PAGE gels shown in Extended Data Figures. **(a)** 10% SDS-PAGE gel for ED Fig. 2a,e showing $KaiC_{RS}$ autophosphorylation over time at 20, 25, 30, and 35 °C. **(b)** 6.5% SDS-PAGE gel for ED Fig. 2c showing autophosphorylation of $KaiC_{SE}$ in the presence of $KaiA_{SE}$ at 30 °C. **(c)** Zn^{2+} Phos-tag^{TM} SDS-PAGE gel for ED Fig. 3e showing autophosphorylation of wild-type $KaiC_{RS}$ and $KaiC_{RS}$-Δcoil at 30 °C. **(d)** 10% SDS-PAGE gel for ED Fig. 3i showing dephosphorylation of $KaiC_{RS}$ at 30 °C. **(e)** 10% SDS-PAGE gel for ED Fig. 6a showing auto-phosphorylation cycle restarts upon regeneration of ATP at 30 °C. **(f)** 10% SDS-PAGE gel for ED Fig. 6b showing autophosphorylation of $KaiC_{RS}$ with and without $KaiB_{RS}$ at 30 °C (with an ATP-recycling system). **(g)** 10% SDS-PAGE gel for ED Fig. 7e showing dephosphorylation of $KaiC_{RS}$ with and without $KaiB_{RS}$ at 30 °C. **(h)** SDS-PAGE gel for ED Fig. 10e showing pull-down assay to measure the complex formation between $KaiB_{RS}$-Tag and wild-type $KaiC_{RS}$, $KaiC_{RS}$-S413E/S414E, or $KaiC_{RS}$-S413A/S414A in the presence of 4 mM ADP or ATP (with an ATP-recycling system). U, S, and D represent unphosphorylated, singly, and doubly phosphorylated protein, respectively.

Pitsawong, Pádua, & Grant et al., 2023

Supplementary Table 1: Codon-optimized DNA sequence for $KaiC_{RS}$ and $KaiB_{RS}$ constructs

Protein	Plasmid	DNA Sequence
$KaiC_{RS}$	pETM-41	ATGGGCATCGGCAAGAGCCCGACCGGCATTCAGGGTTTCGACGAACTGACCCTGGGTGGCCT<br>GCCGACCGGCCGTCCGAGCCTGGTTTGCGGTAGCGCGGGTTGCGGTAAAACCCTGTTCGCGA<br>GCACCTTTCTGATTAACGGCGTGCGTGATCACGGCGAACCGGGTGTTTTCGTGACCTTTGAG<br>GAACGTCCGGAGGACATCGTTAACAACGTGGCGAGCCTGGGTTTCGAACTGGATAAGCTGAT<br>CGAGGAAGAGAAAATCGCGATTGAGCACATTGCGGTTGACCCGAGCGAAGTGGCGGAGATCG<br>GCGACTACGATCTGGAGGGTCTGTTTCTGCGTCTGGAACTGGCGATTGACACCGTTGGTGCG<br>AAGCGTGTGGTTCTGGATACCATCGAAAGCCTGTTCAGCGCGTTTAGCAACCCGGCGATCCT<br>GCGTGCGGAGATTCGTCGTCTGTTCGACTGGCTGAAAGAACGTGGCCTGACCACCGTGATTA<br>CCGCGGAGCGTGGCGATGGTGCGCTGACCCGTCAAGGTCTGGAAGAGTATGTTAGCGACTGC<br>GTGATCCTGCTGGATCACCGTGTTGAGAACCAGATCAGCACCCGTCGTCTGCGTATTGTGAA<br>GTACCGTGGCACCGCGCACGGCACCAACGAATATCCGTTCCTGATCGACACCGATGGCTTTA<br>GCGTTCTGCCGGTGAGCGCGCTGGGTCTGCTGCACCAAGTTCACGAAGAGCGTATTGCGAGC<br>GGTGTGCCGGACCTGGATGCGATGATGGCGGGTGGCGGTTTCTTTCGTGGCAGCAGCATTCT<br>GGTTAGCGGTGTGGCGGGTGCGGGTAAAAGCAGCCTGGCGGCGCACTTTGCTGCGGCGGCGT<br>GCGCGCGTGGCGAGCGTGCGATGTACTTCAGCTTTGAAGAGGCGGCGGATCAGGCGGTTCGT<br>AACATGCGTAGCCTGGGCCTGGACCTGGGTCGTTGGCGTGATGCGGGTCTGCTGCGTTTCAT<br>GGCGACCCGTCCGACCTTTTATAGCCTGGAAATGCACCTGGCGGTTATTCTGCGTGAGGTGA<br>TGCGTTTCGAACCGAGCGTGGTTGTGCTGGACCCGATCAGCGCGTTTACCGAGAGCGGTGAT<br>CGTCTGGAAGTTCAAAGCATGCTGCTGCGTATTGTGGACTTCCTGAAGAACCGTGGCATCAC<br>CGGTATTTTTACCCACCTGGCGCACAGCCAAAACGAGGCGACCACCGATGCGGGCCTGAGCA<br>GCCTGATGGATGGTTGGGTTCTGATGCTGAACCGTGAAGTGAACGGCGAGTTCAACCGTGAA<br>CTGTACCTGCTGAAGGCGCGTGGTATGGCGCACAGCAACCAAGTTCGTGAGTTTCTGATGAG<br>CGATCGTGGTATTAGCCTGCTGCCGCCGCACCTGGGTGAAGGCGGTGCGCTGACCGGCACCG<br>CGCGTAAAGCGGAAGAGGCGCGTCTGCGTCGTGCGGAAATCGAGCGTCAGACCGAGCTGGGT<br>CGTCTGCAGCAACAGATTGAACAACGTCGTCGTCGTGCGCGTGCGCAGATTGAGGCGCTGGA<br>AGCGGAGCTGCAAGCGGAAGAGATCGCGCTGAAGGCGCTGGTTGAGAGCGAGAGCGCGCATG<br>AACGTCAACGTCTGGCGGATGCGGATACCCTGGCGCGTAGCCGTGGCAACGAACGTTTCGCG<br>GACCTGCTGATGAACAAAGGTGAG
$KaiB_{RS}$	pETM-41	ATGGGTCGTCGTCTGGTGCTGTATGTGGCGGGTCAAACCCCGAAAAGCCTGGCGGCGATTAG<br>CAACCTGCGTCGTATCTGCGAGGAAAACCTGCCGGGCCAGTACGAGGTGGAAGTTATCGACC<br>TGAAGCAAAACCCGCGTCTGGCGAAAGAGCACAGCATCGTGGCGATTCCGACCCTGGTGCGT<br>GAACTGCCGGTTCCGATCCGTAAGATTATTGGTGACCTGAGCGATAAAGAACAAGTGCTGGT<br>GAACCTGAAAATGGACATGGAG

		Protein Sequence
$KaiC_{RS}$	pETM-41	<u>*GA</u>MGIGKSPTGIQGFDELTLGGLPTGRPSLVCGSAGCGKTLFASTFLINGVRDHGEPGVFV<br>TFEERPEDIVNNVASLGFELDKLIEEEKIAIEHIAVDPSEVAEIGDYDLEGLFLRLELAIDT<br>VGAKRVVLDTIESLFSAFSNPAILRAEIRRLFDWLKERGLTTVITAERGDGALTRQGLEEYV<br>SDCVILLDHRVENQISTRRLRIVKYRGTAHGTNEYPFLIDTDGFSVLPVSALGLLHQVHEER<br>IASGVPDLDAMMAGGGFFRGSSILVSGVAGAGKSSLAAHFAAAACARGERAMYFSFEEAADQ<br>AVRNMRSLGLDLGRWRDAGLLRFMATRPTFYSLEMHLAVILREVMRFEPSVVVLDPISAFTE<br>SGDRLEVQSMLLRIVDFLKNRGITGIFTHLAHSQNEATTDAGLSSLMDGWVLMLNREVNGEF<br>NRELYLLKARGMAHSNQVREFLMSDRGISLLPPHLGEGGALTGTARKAEEARLRRAEIERQT<br>ELGRLQQQIEQRRRRARAQIEALEAELQAEEIALKALVESESAHERQRLADADTLARSRGNE<br>RFADLLMNKGE
$KaiB_{RS}$	pETM-41	<u>*GA</u>MGRRLVLYVAGQTPKSLAAISNLRRICEENLPGQYEVEVIDLKQNPRLAKEHSIVAIPT<br>LVRELPVPIRKIIGDLSDKEQVLVNLKMDME

*<u>*GA*</u> are residues that are part of the after TEV protease recognition site and remain after cleavage. Residue numbering of both $KaiC_{RS}$ and $KaiB_{RS}$ start counting after residues *GA*.

Supplementary Table 2: Primers for site-directed mutagenesis and sequencing of $KaiC_{RS}$

Mutation		Primer
$KaiC_{RS}$ S413E	5' GCGACCACCGATGCGGGCCTGGAAAGCCTGATGGATGGTTGGGTTC 3'	Forward
	5' GAACCCAACCATCCATCAGGCTTTCCAGGCCCGCATCGGTGGTCGC 3'	Reverse
$KaiC_{RS}$ S413E/S414E	5' GCGACCACCGATGCGGGCCTGGAAGAACTGATGGATGGTTGGGTTC 3'	Forward
	5' GAACCCAACCATCCATCAGTTCTTCCAGGCCCGCATCGGTGGTCGC 3'	Reverse
$KaiC_{RS}$ S413A/S414A	5' GAGGCGACCACCGATGCGGGCCTGGCGGCGCTGATGGATGGTTGGGTTCTGATG 3'	Forward
	5' CATCAGAACCCAACCATCCATCAGCGCCGCCAGGCCCGCATCGGTGGTCGCCTC 3'	Reverse
$KaiC_{RS}$ E62Q/E63Q	5' CACGGCGAACCGGGTGTTTTCGTGACCTTTCAGCAACGTCCGGAGGACATCGTTAACAAC 3'	Forward
	5' GTTGTTAACGATGTCCTCCGGACGTTGCTGAAAGGTCACGAAAACACCCGGTTCGCCGTG 3'	Reverse
$KaiC_{RS}$ E302Q/E303Q	5' GTGCGATGTACTTCAGCTTTCAACAGGCGGCGGATCAGGC 3'	Forward
	5' GCCTGATCCGCCGCCTGTTGAAAGCTGAAGTACATCGCAC 3'	Reverse
$KaiC_{RS}$-Δcoil	5' GCGGAAGAGGCGCGTCTGCGTCGTGCGTAAATCGAGCGTCAGACCGAGCTGGGTCG 3'	Forward
(residues 1–489)	5' CGACCCAGCTCGGTCTGACGCTCGATTTACGCACGACGCAGACGCGCCTCTTCCGC 3'	Reverse
$KaiCI_{RS}$	5' GACACCGATGGCTTTAGCGTTCTGTAGGTGAGCGCGCTGGGTCTGCTGCACCAA 3'	Forward
(residues 1–230)	5' TTGGTGCAGCAGACCCAGCGCGCTCACCTACAGAACGCTAAAGCCATCGGTGTC 3'	Reverse
*$KaiCI_{RS}$ E62Q/E63Q	5' CACGGCGAACCGGGTGTTTTCGTGACCTTTCAGCAACGTCCGGAGGACATCGTTAACAAC 3'	Forward
	5' GTTGTTAACGATGTCCTCCGGACGTTGCTGAAAGGTCACGAAAACACCCGGTTCGCCGTG 3'	Reverse

Reading area	Primer sequence
From MBP-Val^{260} to $KaiC_{RS}$-Gly^{160}	5' GGTGTAACGGTACTGCCGACC 3'
From Ala^{140} to Lys^{441}	5' GCGATTGACACCGTTGGT 3'
From Leu^{399} to Ser^{568}	5' AAGCATGCTGCTGCGTATTG 3'

*Using $KaiCI_{RS}$ as a template for site-directed mutagenesis
