# Supplementary material for: From primordial clocks to circadian oscillators
Source: Nature. 2023 Mar 22;616(7955):183–9. doi: 10.1038/s41586-023-05836-9 (PMC10076222; doi:10.1038/s41586-023-05836-9)
Supplement: Supplementary file 2 — Reporting Summary [file 41586_2023_5836_MOESM2_ESM.pdf]

nature portfolio

Corresponding author(s): Dorothee Kern

Last updated by author(s): 01/21/2023

Reporting Summary

Nature Portfolio wishes to improve the reproducibility of the work that we publish. This form provides structure for consistency and transparency in reporting. For further information on Nature Portfolio policies, see our Editorial Policies and the Editorial Policy Checklist.

Statistics

For all statistical analyses, confirm that the following items are present in the figure legend, table legend, main text, or Methods section.

n/a	Confirmed	
☐	☒	The exact sample size (*n*) for each experimental group/condition, given as a discrete number and unit of measurement
☐	☒	A statement on whether measurements were taken from distinct samples or whether the same sample was measured repeatedly
☒	☐	The statistical test(s) used AND whether they are one- or two-sided<br>*Only common tests should be described solely by name; describe more complex techniques in the Methods section.*
☒	☐	A description of all covariates tested
☒	☐	A description of any assumptions or corrections, such as tests of normality and adjustment for multiple comparisons
☐	☒	A full description of the statistical parameters including central tendency (e.g. means) or other basic estimates (e.g. regression coefficient) AND variation (e.g. standard deviation) or associated estimates of uncertainty (e.g. confidence intervals)
☒	☐	For null hypothesis testing, the test statistic (e.g. *F*, *t*, *r*) with confidence intervals, effect sizes, degrees of freedom and *P* value noted<br>*Give P values as exact values whenever suitable.*
☒	☐	For Bayesian analysis, information on the choice of priors and Markov chain Monte Carlo settings
☒	☐	For hierarchical and complex designs, identification of the appropriate level for tests and full reporting of outcomes
☒	☐	Estimates of effect sizes (e.g. Cohen's *d*, Pearson's *r*), indicating how they were calculated

Our web collection on statistics for biologists contains articles on many of the points above.

Software and code

Policy information about availability of computer code

Data collection	All data collection was performed using the software provided with the respective instruments:<br>- Cryo-EM: SerialEM v3.6<br>- Fluorescence (anisotropy): FluorEssence v3.5 (HORIBA Scientific)<br>- SDS page gels scannner: ChemiDoc Imager v 6.0.1 (Bio-Rad)<br>- TLC plate scanner: Amersham Typhoon v5 (GE Healthcare)
Data analysis	Most of the software used for the analysis described in this paper are freely/publicly available:<br>- X-ray Crystallographic data was analyzed using COOT 0.9.8.1, programs in the PHENIX 1.20.1-4487 software suite (e.g., MRage and Phaser). Additional software packages include Zanuda available in the software package CCP4 7.4, Achesym (web server, no version information available), SamCC-Turbo 0.0.2, PyMOL 2.6.0, and CAVER 3. The Supplementary Video was created in part by using UCSF Chimera 1.15.<br>- cryo-EM data was analyzed using cisTEM 2.0.0-alpha, COOT 0.9.8.1, and PHENIX 1.20.1-4487.<br><br>Commercially available software used:<br>- KinTek Explorer 10 for the analysis of kinetic experiments<br>- Kaleidagraph 4.5.3 (Synergy) for the fitting/plotting of all biochemical experiments<br>- Gel densitometry: Image Lab v 6.0.1 (Bio-Rad)<br>- TLC plates scanner: ImageQuant TL 7.0 software

For manuscripts utilizing custom algorithms or software that are central to the research but not yet described in published literature, software must be made available to editors and reviewers. We strongly encourage code deposition in a community repository (e.g. GitHub). See the Nature Portfolio guidelines for submitting code & software for further information.

Data

Policy information about availability of data

All manuscripts must include a data availability statement. This statement should provide the following information, where applicable:

- Accession codes, unique identifiers, or web links for publicly available datasets
- A description of any restrictions on data availability
- For clinical datasets or third party data, please ensure that the statement adheres to our policy

Data availability
Structure factors and refined models obtained using X-ray crystallography are deposited in the Protein Data Bank (PDB) under accession codes 8dba (wild-type KaiCRS) and 8db3 (KaiCRS-Δcoil).

Cryo-EM maps and refined models are deposited in the Electron Microscopy Data Bank (EMDB) and Protein Data Bank (PDB), respectively. The composite map and model for the KaiCRS-S413E/S414E dodecamer reconstruction are submitted under entries EMD-29505 and 8fwi, respectively. The composite map and model for the KaiCRS-S413E/S414E:KaiBRS dodecamer reconstruction are submitted under entries EMD-29506 and 8fwj, respectively. The focused KaiCRS-S413E/S414E hexamer refinement map is available under accession EMD-29507 and the focused KaiCRS-S413E/S414E:KaiBRS hexamer refinement map is available under accession EMD-29508. The full KaiCRS-S413E/S414E dodecamer refinement is available under accession EMD-29509 and the full KaiCRS-S413E/S414E:KaiBRS dodecamer refinement is available under accession EMD-29510.

Other data sets used are all publicly available in public community/discipline-specific repositories: PDB 5jwq, PDB 1w0j, PDB 1tf7, PDB 7s65, the accession codes for protein sequences used the sequence alignments/phylogeny are listed in Supplementary Datasets 1 and 2.

Human research participants

Policy information about studies involving human research participants and Sex and Gender in Research.

Reporting on sex and gender	N/A
Population characteristics	N/A
Recruitment	N/A
Ethics oversight	N/A

Note that full information on the approval of the study protocol must also be provided in the manuscript.

Field-specific reporting

Please select the one below that is the best fit for your research. If you are not sure, read the appropriate sections before making your selection.

☒ Life sciences ☐ Behavioural & social sciences ☐ Ecological, evolutionary & environmental sciences

For a reference copy of the document with all sections, see nature.com/documents/nr-reporting-summary-flat.pdf

Life sciences study design

All studies must disclose on these points even when the disclosure is negative.

Sample size	The sample size has been described in the figure legends where applicable. No statistical method has been used to predetermine the sample size. Experiments were typically repeated in triplicates independently to ensure the robustness of the conclusions.
Data exclusions	For the phylogenetic tree, sequences with a sequence homology above 90% were excluded from the final analysis. For all other experiments no data were excluded from the analyses.
Replication	The replicate number for experiments is described in the figure legends. Typically, experiments were repeated as biological triplicates to ensure the robustness of the conclusions. All attempts at replication were successful.
Randomization	Not applicable as there were no groups to be allocated.
Blinding	No allocation into groups was performed (see above), so "blinding" of investigators is not applicable.

Reporting for specific materials, systems and methods

We require information from authors about some types of materials, experimental systems and methods used in many studies. Here, indicate whether each material, system or method listed is relevant to your study. If you are not sure if a list item applies to your research, read the appropriate section before selecting a response.

Materials & experimental systems

n/a	Involved in the study
☒	☐ Antibodies
☒	☐ Eukaryotic cell lines
☒	☐ Palaeontology and archaeology
☒	☐ Animals and other organisms
☒	☐ Clinical data
☒	☐ Dual use research of concern

Methods

n/a	Involved in the study
☒	☐ ChIP-seq
☒	☐ Flow cytometry
☒	☐ MRI-based neuroimaging
